# Development of an NGS-Based Workflow for Improved Monitoring of Circulating Plasmids in Support of Risk Assessment of Antimicrobial Resistance Gene Dissemination

**DOI:** 10.3390/antibiotics9080503

**Published:** 2020-08-11

**Authors:** Bas Berbers, Pieter-Jan Ceyssens, Pierre Bogaerts, Kevin Vanneste, Nancy H. C. Roosens, Kathleen Marchal, Sigrid C. J. De Keersmaecker

**Affiliations:** 1 Transversal Activities in Applied Genomics, Sciensano, 1050 Brussels, Belgium; Bas.Berbers@Sciensano.be (B.B.); Kevin.Vanneste@Sciensano.be (K.V.); Nancy.Roosens@Sciensano.be (N.H.C.R.); 2 Department of Information Technology, IDLab, Ghent University, IMEC, 9052 Ghent, Belgium; Kathleen.Marchal@UGent.be; 3 Bacterial Diseases, Sciensano, 1050 Brussels, Belgium; Pieter-Jan.Ceyssens@Sciensano.be; 4 National Reference Center for Antimicrobial Resistance in Gram-Negative Bacteria, CHU UCL Namur, 5530 Yvoir, Belgium; Pierre.Bogaerts@uclouvain.be; 5 Department of Plant Biotechnology and Bioinformatics, Ghent University, 9052 Ghent, Belgium; 6 Department of Genetics, University of Pretoria, Pretoria 0083, South Africa

**Keywords:** plasmids, antimicrobial resistance, conjugation, DNA extraction, next-generation sequencing, MiSeq, MinION, Flongle, hybrid assembly, surveillance, mobile elements

## Abstract

Antimicrobial resistance (AMR) is one of the most prominent public health threats. AMR genes localized on plasmids can be easily transferred between bacterial isolates by horizontal gene transfer, thereby contributing to the spread of AMR. Next-generation sequencing (NGS) technologies are ideal for the detection of AMR genes; however, reliable reconstruction of plasmids is still a challenge due to large repetitive regions. This study proposes a workflow to reconstruct plasmids with NGS data in view of AMR gene localization, i.e., chromosomal or on a plasmid. Whole-genome and plasmid DNA extraction methods were compared, as were assemblies consisting of short reads (Illumina MiSeq), long reads (Oxford Nanopore Technologies) and a combination of both (hybrid). Furthermore, the added value of conjugation of a plasmid to a known host was evaluated. As a case study, an isolate harboring a large, low-copy *mcr-1*-carrying plasmid (>200 kb) was used. Hybrid assemblies of NGS data obtained from whole-genome DNA extractions of the original isolates resulted in the most complete reconstruction of plasmids. The optimal workflow was successfully applied to multidrug-resistant *Salmonella* Kentucky isolates, where the transfer of an ESBL-gene-containing fragment from a plasmid to the chromosome was detected. This study highlights a strategy including wet and dry lab parameters that allows accurate plasmid reconstruction, which will contribute to an improved monitoring of circulating plasmids and the assessment of their risk of transfer.

## 1. Introduction

Antimicrobial resistance (AMR) genes are inherently present in bacteria. However, the (mis)use of antibiotics increased the global prevalence of AMR; as a result, it has become one of the most prominent public health threats [1]. Plasmids are mobile genetic elements that can carry various genes beneficial for the survival of bacteria, including AMR genes. Plasmids are highly diverse in their genetic content, size (1–1000 kb) and copy number (1−500). Bacteria can exchange plasmids between isolates even across the species or phyla barriers, also called horizontal gene transfer (HGT) [2,3,4,5,6]. This transfer of plasmids facilitates the transmission of accompanying AMR genes, making plasmid detection and reconstruction of the utmost importance to understand the dissemination of AMR genes and to improve risk assessment.

Due to the globalization of the modern world, plasmids with AMR genes of clinical relevance can disseminate in a short time span [3,7,8]. There has been an increase of extended-spectrum β-lactamase (ESBL)-producing Enterobacterales (containing variants of the *bla*_TEM_, *bla*_SHV_ and *bla*_CTX-M_ ESBL genes) reported in Europe caused by specific clones and plasmids [9]. Moreover, the carbapenemase genes encoding for OXA-48, KPC, VIM, NDM and IMP have been increasingly detected, and while carbapenemase-producing Enterobacterales (CPE) used to only be reported in hospital settings, nowadays CPE are also detected in community and environmental samples in Europe [10]. Moreover, these carbapenemase genes have also been increasingly reported on plasmids [11]. Of extra concern is that the described plasmids are often carrying multiple AMR genes, making treatment of infections caused by bacteria carrying these plasmids even more troublesome and sometimes leaving only colistin as a last resort antimicrobial. Unfortunately, the *mcr-1* gene conferring resistance to the last-line antibiotic colistin has recently been detected on a plasmid in China [12]. Shortly afterwards, *mcr-1* was detected worldwide with co-localization of ESBL genes on a plasmid [13,14,15,16]. Furthermore, the European Food Safety Authority (EFSA) reported that food-borne infections are becoming harder to treat as a consequence of the increase in AMR prevalence [17]. However, the data on circulating plasmids are only slowly increasing, and in particular, detailed knowledge on the plasmid structure and rearrangements is severely lacking, which can hinder risk assessment [18].

There are several techniques to detect AMR genes and plasmids, of which PCR is a commonly used one [19,20,21,22,23]. (q)PCR allows to indicate the presence of a plasmid or AMR gene, with one targeted PCR test per AMR gene/plasmid locus to be identified [20,21]. However, the determination whether the AMR gene is localized on a plasmid necessitates additional experiments including time-consuming Southern blotting [24] or plasmid transfer followed by further analyses [25]. With next-generation sequencing (NGS), multiple aspects such as the complete resistance profile, including the AMR gene sequence, and plasmid elements can be determined simultaneously, without a priori knowledge [22,23]. Short-read sequencing technologies showed to have high accuracy in detecting the AMR genes, with some reports stating high concordance with phenotypic results in Enterobacterales [26]. However, plasmids often contain large repetitive regions that cannot be bridged by short sequencing reads [27,28,29,30], resulting in incomplete contigs of which the origin, chromosome or plasmid, is uncertain. If the AMR genes are localized on these incomplete contigs, it is uncertain whether they are localized on a plasmid (higher risk of transfer). Similarly, there are insertion sequences (IS) of 600–7900 bp which contain AMR genes and are flanked by short direct repeats [31]. IS can be present on either the chromosome or a plasmid; however, their location cannot always be resolved with short-read sequencing. Since the introduction of third-generation sequencers (Oxford Nanopore Technologies and Pacific Biosciences (PacBio)), it is possible to generate long sequencing reads (>1000 bp) in high throughput that are able to bridge these repetitive regions [30,32,33]. However, these long reads also contain more errors (10–20% error rate) compared to short reads, which can hinder AMR gene prediction. Others have shown that by using short reads in combination with long reads, it is possible to reconstruct full genomes, including plasmids [30,33,34,35,36]. Aside from the higher cost of sequencing with two technologies, another issue is that long-read sequencing requires high amounts of input DNA of high molecular weight [37]. These restrictions might not always be compatible with the current DNA extraction protocols routinely used for surveillance. Therefore, to obtain the most optimal plasmid reconstruction with NGS, there are multiple parameters to consider, both at the wet and dry lab level. However, specific guidelines on what would be the best approach to take for efficient plasmid reconstruction are still missing.

One parameter to consider is the input DNA material to be used. As a starting isolate, the plasmid-containing original strain could be used, or the plasmid could first be transferred to a lab-host recipient with a known chromosome sequence [38]. Although a transfer would take more hands-on time compared to working directly with the original (clinical, food, environmental) isolates, the benefit would be that as the complete chromosome sequence is already known, reads matching to this sequence could be filtered out during the bioinformatics analysis, in theory only leaving plasmid reads in the data set for plasmid reconstruction [22]. Alternatively to this in silico approach to retrieve plasmid reads, a plasmid DNA extraction method could be used instead of a method targeting the total genome [39,40], thereby leaving only plasmid DNA as input for the NGS approach. Most plasmid DNA extractions are based on the difference in size between chromosomal and plasmid DNA and start with an alkaline lysis [41] that denatures all DNA. Shorter DNA fragments (plasmids) renature more quickly, and this enables them to be separated from the chromosomal DNA by several methods [42]. However, this is not always successful, as some plasmids might be lost during extraction, or chromosomal DNA may remain in the final extract. It has been reported that column-based plasmid DNA extractions have difficulties with extracting plasmids >150 kb in size [43]. Additionally, an insufficient amount of plasmid DNA might be obtained [44,45], which, as mentioned, might be an issue for long-read technologies (Nanopore or PacBio) which require >500 ng of high-molecular-weight input DNA depending on the library preparation protocol [37,46]. Nevertheless, plasmid DNA extractions have been successfully used with long-read sequencing for the reconstruction of plasmids [33,36]. However, a systematic comparison on the effect of different DNA extraction protocols on plasmid reconstruction has not yet been reported.

Another parameter to consider is the sequencing technology. For short-read sequencing, Illumina has been by far the most used. However, as elaborated above, short reads only might be insufficient for plasmid reconstruction. There are currently two accessible long-read sequencing technologies, i.e., from Nanopore and PacBio. While the error rate of the PacBio technology is lower [47], Nanopore technology produces a higher yield and longer reads [48]. Moreover, an important advantage of the MinION flow cells from Nanopore is that they are accessible to more labs due to the lower initial investment and size [47]. Additionally, the MinION flow cells are more cost-efficient, which is important to consider when a large number of isolates in routine analyses or surveillance need to be sequenced. Recently, Nanopore released the Flongle, which is a smaller version of the MinION, making it ideal for sequencing a single bacterial isolate. However, the Flongle has not yet been tested for the reconstruction of plasmids.

The last parameter to consider is the bioinformatic analysis. After sequencing, the reads are usually trimmed and filtered [49]. Then, the overlap between sequencing reads (assembly) is used to reconstruct the genomic structures, or the reads are directly used in alignments to known plasmid sequences. The overlap between sequencing reads is determined automatically by software (assembly tools) that makes use of only short or long reads, or a combination of both sequencing reads (hybrid). As aforementioned, using only short-read sequencing for reconstruction of plasmids is often insufficient due to the repetitive regions in plasmids [27,28,29,30], and therefore long-read sequencing is needed. In routine testing, however, cost and accuracy of AMR detection and full plasmid reconstruction have to be taken into account. Nevertheless, it has not been systematically evaluated yet whether long-read only or hybrid assemblies are more suitable for this purpose. Subsequently, more information can be retrieved from the assemblies by comparing them to databases for AMR genes [50,51] or plasmid replicons/sequences [52,53,54].

Although there have been other studies that compared different (plasmid) DNA extraction methods and/or assemblies [44,55,56,57,58,59], these studies only focused on one of the aforementioned parameters. Moreover, for some of these studies, the plasmid reconstruction was not the main focus, and therefore the completeness or AMR profile of the reconstructed plasmid(s) was not evaluated. As the setup/focus of these studies was very different, involving, e.g., different isolates and plasmids, it is difficult to compare the results in view of a systematic evaluation. Therefore, this study aimed to determine the effect of the above-described parameters on the NGS results and the reconstruction of plasmids in the context of AMR monitoring. In view of proposing an optimal workflow covering both the wet and dry lab, the reconstruction of an *mcr-1* plasmid from several whole-genome and plasmid DNA extracts with only MiSeq or MinION data and a combination of both (hybrid) were compared. Both the original isolate and the corresponding transconjugant were used. Furthermore, the newly released Flongle flow cell from Nanopore was used for sequencing, and the produced data were evaluated for the feasibility of plasmid reconstruction. Finally, the obtained workflow was applied to multidrug-resistant *Salmonella* Kentucky isolates for which it had been impossible to determine with solely short-read sequencing whether the clinically relevant ESBL genes were localized on a plasmid or on the chromosome.

## 2. Results

### 2.1. Development of an Optimized Workflow for Plasmid Reconstruction Using NGS Data

**Figure 1:**
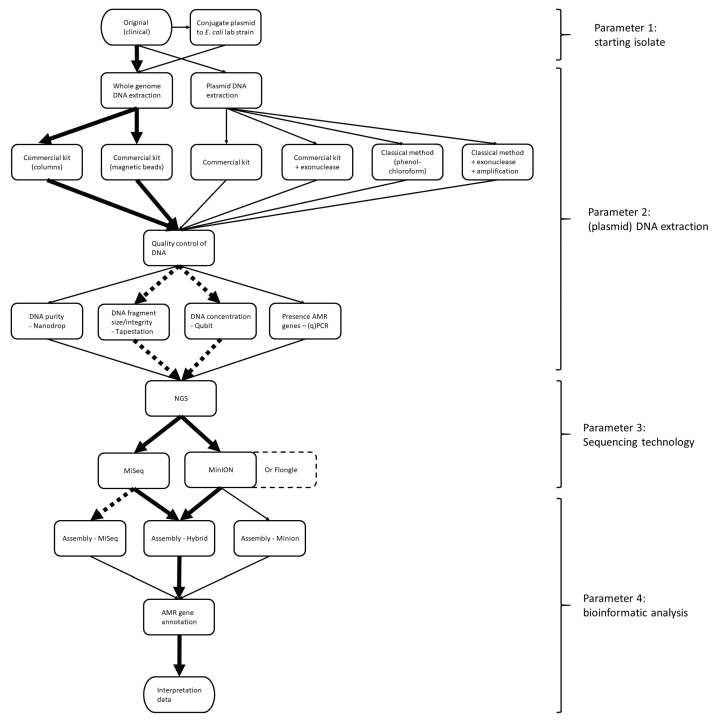
Overview of workflow to reconstruct plasmids with next-generation sequencing and the parameters that were tested. The arrows in bold represent the parameters that were found to be most suitable for reconstructing plasmids. The dashed, bold arrows represent parameters that are optional for the workflow but were determined to be more useful than the nonbold arrows. The box encapsulated in a dashed line represents a parameter that is very similar to the one it is adjacent to.

In Figure 1, the workflow used for this study is visualized, wherein we focused on comparing the nature of the starting isolate (original isolate or conjugated lab-strain) and the DNA extraction method (whole-genome or plasmid). During the comparison, the impacts of the sequencing technology (short- or long-read sequencers) and the bioinformatic analysis (assemblies from one technology or a combination of both) on plasmid reconstruction were also addressed. Additionally, the latter two parameters were further evaluated by comparing the accuracy of the predicted AMR genes on the identified contigs.

#### 2.1.1. Assessing the Nature of the Starting Isolate

**Figure 2:**
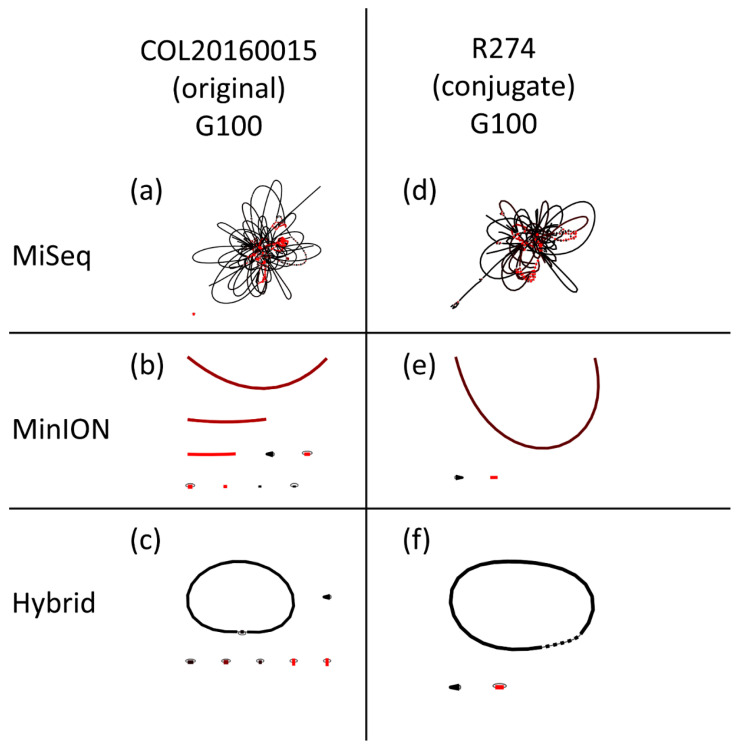
Visualizations of the assembly graphs with Bandage of the MiSeq, MinION and hybrid assemblies performed on the whole-genome DNA extractions (Genomic Tip 100) of the original *E. coli* COL20160015 (**a**–**c**) and the conjugate R274 (**d**–**f**). Each line represents a contig, and a red line represents a contig occurring in higher coverage compared to the other contigs within the same assembly. The thinner black lines are the most likely paths between contigs.

First, the impact of the choice of the starting isolate was evaluated, i.e., we assessed whether conjugation of the plasmid to a lab strain with known genome sequence facilitates plasmid reconstruction. To this end, the isolate COL20160015 was used, which is a clinical *Escherichia coli* with an *mcr-1*-harboring plasmid (>200 kb) (extr-1; Table 1). This *mcr-1*-containing plasmid was transferred into a lab strain *E. coli* yielding conjugate R274 (extr-2; Table 1). Short (MiSeq) and long (MinION) read sequencing was performed on genomic DNA extracts of both isolates. Before doing any filtering of the chromosomal reads of the conjugated isolate, the assemblies of each sequencing technology separately and a combination of both (hybrid) (Figure 2) and the AMR profile (Tables S1 and S2) were compared as such.

The MiSeq assemblies were very fragmented (>100 contigs), and it was not possible to close the chromosome nor the plasmid. Moreover, all contigs showed connections in the assembly graphs (Figure 2a,d), and therefore it was not possible to attribute the AMR genes to either the chromosome or a plasmid. The assemblies made with only MinION reads (Figure 2b,e) improved the assembly (less fragmented, higher N50). However, it was not possible to circularize every contig, making the origin of the contig ambiguous. Nonetheless, large contigs above 500 kb are likely of chromosomal origin, because natural plasmids of this size are rarely reported. With hybrid assemblies (Figure 2c,f), it was possible to reconstruct the *mcr-1*-positive plasmid in one circular contig of 238,070 bp. In the conjugated isolate R274 there was an additional circular contig of 113 kb that was confirmed with BLAST to be part of a repetitive region in the chromosome of the lab-strain *E. coli*. In the assembly graph of *E. coli* COL20160015, there is a 20 kb contig connected to the chromosome at both 3′ and 5′ sides (bubble), which also corresponds to a repetitive region. Interestingly, the *E. coli* COL20160015 hybrid assembly showed five circular plasmids and one circular phage (determined by comparison to NCBI and PHASTER databases). The plasmids ranged from 3 to 238 kb, and the smaller plasmids were present in high copy number (16–20×), while the large plasmids showed low copy numbers (1–4×) based on the read coverage. Of these six mobile genetic elements, only the largest plasmid (238 kb) was present in the conjugated strain. The four plasmids that were not conjugated contained AMR genes conferring resistance to clinically relevant antibiotics, e.g., aminoglycoside, beta-lactam, macrolide, phenicol, quinolone, rifampicin, sulfonamide and trimethoprim antibiotics (Table S1).

In theory, the advantage of first conjugating the plasmid to a host strain with known genome sequence would be that all reads from the chromosome of the conjugated isolate (R274) could be removed first, followed by an assembly of the remaining plasmid reads. However, in practice, it turned out that mapping all reads to the reference genome of the host strain (accession number: CP000948.1) followed by hybrid assemblies with all the unmapped MiSeq and MinION reads resulted in an incomplete (noncircular) contig of 237,182 bp in size. Nevertheless, this incomplete, linear contig contained all expected AMR genes and the plasmid replicon (Table S3). Similarly, all the expected AMR genes were also present in the assemblies made with only the unmapped MiSeq reads or the MinION reads (Table S3). Moreover, the assembly made with only the unmapped MiSeq reads was less fragmented than the MiSeq assemblies made with all reads.

By comparing the reference genome of the host strain with the complete circular plasmid contig (found when performing a hybrid assembly of all MiSeq and MinION reads of R274) using BLAST, a region of 769 bp at position 146,460–147,228 in the plasmid was detected that was also present in the reference genome. Reads mapping to this location in the plasmid were likely removed during the filtering step with the reference genome, which resulted in the assembly of an incomplete plasmid contig.

The *mcr-1* plasmid reconstructed from *E. coli* COL20160015 and R274 had been described before in an *E. coli* in Germany in 2017 (LT599829.1), as determined by BLAST analysis.

#### 2.1.2. Assessing DNA Extraction Methods

As no added-value for conjugation in plasmid reconstruction could be highlighted, the evaluation of parameter 2, i.e., whole-genome or plasmid DNA extraction, was performed on an original isolate. A *Salmonella enterica* isolate that was previously sequenced with only MiSeq was selected (S15BD05371). This isolate had a less complex AMR and plasmid profile (only an IncHI2 replicon detected), and the incomplete contigs indicated that it likely contained one large *mcr-1* plasmid (>200 kb). An isolate with a large plasmid was chosen as it is known that large plasmids cause the most problems in plasmid extractions and assemblies.

Several DNA extraction protocols were tested, some targeting total genomic DNA, others specifically targeting plasmid DNA (extr-3–8; Table 1). The Genomic Tip 100 (extr-3) was chosen based on the recommendation for Nanopore sequencing for the extraction of genomic DNA with high molecular weight. The automated bead MagCore genomic DNA extraction (extr-4) was evaluated, as it is more adaptable to a routine setting, requiring less hands-on time and being less expensive. For plasmid extraction, a commercial kit (G500, extr-5) and a classical plasmid DNA extraction protocol (phenol, extr-7) were selected. In addition, extended protocols were tested for the plasmid extractions, including an exonuclease treatment to digest contaminating chromosomal DNA (G500-exo and phenol-ampli, extr-6 and extr-8) and an amplification step of the DNA using the REPLI-g kit (phenol-ampli, extr-8) to increase the final DNA concentration.

To evaluate the efficiency of each of the protocols, the purity, concentration, DNA integrity (DIN), fragment size and presence of plasmid versus chromosomal DNA were determined for each DNA extract before sequencing (Table S4). Based on the qPCR results, it was observed that the plasmid DNA extractions phenol (extr-7) and phenol-ampli (extr-8) contained a higher proportion of plasmid DNA. The concentration of the DNA extract G500-exo (extr-6) was very low and even below the detection limit of the measuring method (1 ng/µL). Therefore, this extract (extr-6) was not loaded on the MinION flow cell and was excluded from further analyses. The purity, DNA integrity and fragment size of the MagCore DNA extract (extr-4) were not within the Nanopore specifications. However, it was loaded on the flow cell, as the concentration was sufficiently high according to the recommendations of Nanopore [37].

**Figure 3:**
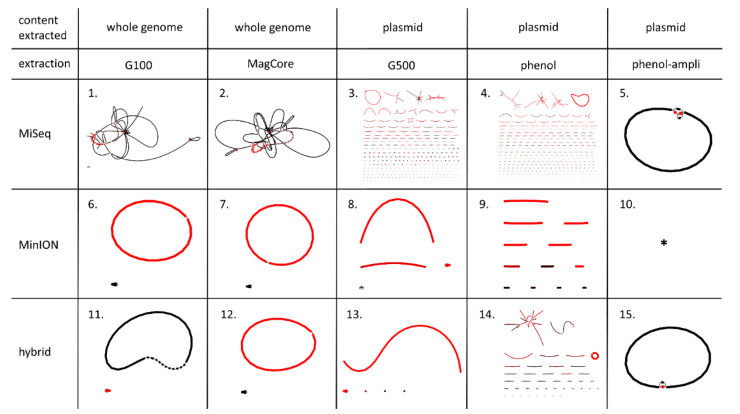
Visualization with Bandage of the MiSeq, MinION and hybrid assemblies performed on the whole-genome and plasmid DNA extractions (G100, MagCore, G500, phenol and phenol-ampli) of *Salmonella* isolate S15BD05371 (extractions 3–5 and 7–8 (Table 1), assemblies 1–15 (Table 2)). Each line represents a contig, and a red line is in higher coverage compared to the other contigs within the same assembly. * There was not enough MinION read coverage to complete the assembly of phenol-ampli with only MinION reads.

MiSeq and MinION sequencing were performed on DNA extracts 3–5 and 7–8 (Table 1). Subsequently, assemblies consisting of solely MiSeq or MinION reads and a combination of both (hybrid) were performed (Figure 3, Table 2). The most ideal assembly of *Salmonella* S15BD05371 would have two circular connected components without dead ends for a whole-genome DNA extract or one such component for a plasmid DNA extract. A dead end is an erroneous structure in an assembly where a contig shows no connection to itself or other contigs on its 5′ or 3′ side; this is usually caused by low sequencing coverage.

The MiSeq assemblies (as-1–5; Table 2) yielded much more contigs than both the MinION (as-6–10; Table 1) and hybrid assemblies (as-11–15; Table 1), except for the MiSeq assembly from the phenol-ampli extract (as-5). The only MiSeq assemblies where there were no dead ends were assemblies 2 (MagCore) and 5 (phenol-ampli).

The MinION assemblies originating from plasmid extracts (as-8–9) contained more contigs than the MinION assemblies from whole-genome extracts (as-6–7). It should however be noted that the MinION flow cell did not generate enough reads for assembly 10 (phenol-ampli) to complete the assembly. All plasmid DNA extractions except for phenol-ampli (extr-7) yielded many incomplete contigs. With BLAST, it was suggested that they were of chromosomal origin, but due to the small size of the contigs this was not very reliable (Table S5). However, by comparing these incomplete contigs to the whole-genome hybrid assemblies, they were determined to be of chromosomal origin.

All assemblies from the whole-genome DNA extracts (Genomic Tip 100 and MagCore) resulted in the ideal number of connected components, except for the MiSeq-only assembly from MagCore (as-2). For the plasmid DNA extractions, only assemblies 5 and 15 (phenol-ampli) produced the ideal number of connected components. Moreover, the only assemblies from plasmid extracts that produced no dead ends were also assemblies 5 and 15 (phenol-ampli), while there were no dead ends in any of the assemblies from whole-genome extracts.

While there were several assemblies that contained the *mcr-1* plasmid in one contig, it was smaller in size for the MinION-only assemblies (as-6–8) than for the hybrid assemblies (as-11–14). The reconstructed *mcr-1* plasmids of the hybrid assemblies were identical in sequence structure (Figure S1). Within the hybrid assemblies, only assembly 15 could not produce the *mcr-1* plasmid in a single contig (although it was still present as one connected component) due to a lower amount of MinION reads.

**Figure 4:**
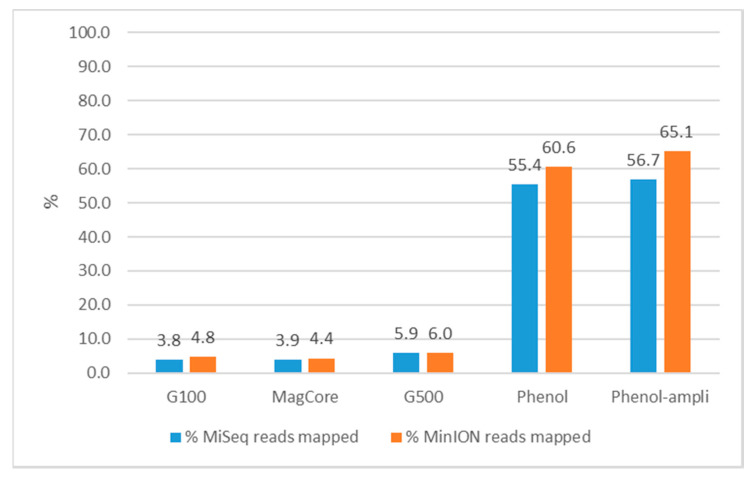
Percentage of MiSeq and MinION reads from the G100 (extr-3), MagCore (extr-4), G500 (extr-5), phenol (extr-7) and phenol-ampli (extr-8) DNA extractions of *Salmonella* isolate S15BD05371 mapped to the complete plasmid sequence.

Furthermore, all MiSeq and MinION reads were mapped to the complete plasmid sequence of *Salmonella* isolate S15BD05371 (retrieved from the hybrid assemblies) (Figure 4). In correspondence with the qPCR results (Table S4), the phenol (extr-7) and phenol-ampli (extr-8) DNA extractions had a higher percentage of the reads which mapped to the plasmid. Even though only 3–6% of the reads mapped to the plasmid in the whole-genome DNA extracts (extr-3, -4), this was still sufficient to completely reconstruct the plasmid with hybrid assemblies. For the Genomic tip 500 extraction with plasmid buffers (extr-5), as already indicated by qPCR (Table S4), the plasmid extraction was unsuccessful as the distribution of plasmid reads was more similar to that of the whole-genome DNA extractions.

By comparing the *mcr-1* plasmid sequence from the hybrid assemblies to the NCBI nucleotide database, it was determined that this sequence had not been described before.

#### 2.1.3. Accuracy of AMR Gene Prediction

In the previous sections, there were already comparisons made between the sequencing technologies and the downstream analyses. However, additional evaluations were done to determine how these parameters affected the accuracy of predicting AMR genes from the resulting assemblies of each sequencing technology separately and a combination of both (hybrid).

In Table 3, the ResFinder, PlasmidFinder and PointFinder results of the MiSeq, MinION and hybrid assemblies from the whole-genome G100 extraction of *Salmonella* S15BD05371 (extr-3 and as-1, -6 and -11) are shown, because these had the most complete resistance profile (chromosome and plasmid). ResFinder results obtained with the other DNA extracts were similar and are shown in the Supplementary Materials (Tables S6–S9).

The plasmid of *Salmonella* S15BD05371 (as-11) carried the genes *aac(3)-IV, aadA2b, aph(4)-Ia, bla*_TEM-1B_*, mcr-1.1, lnu(F), qnrS1, sul3, tet(A)* and *dfrA12*, which theoretically confer resistance to aminoglycoside, beta-lactam, colistin, macrolide, quinolone, sulfonamide, tetracycline and trimethoprim antibiotics. Moreover, the chromosome of *Salmonella* S15BD05371 contained the genes *aac(6′)-Iaa* and *tet(B)*, which also confer resistance to aminoglycoside and tetracycline antibiotics. These results correspond to the tested phenotypical resistance (Table 4). Therefore, the genes detected in the hybrid assembly are considered as the expected AMR genes. 

In the MiSeq assembly (as-1), all expected genes showed high identity percentages. However, the AMR genes were spread out over seven contigs, which showed connections to chromosomal contigs in the assembly graph (Figure 3). Besides the *mcr-1* gene, none of the AMR genes were located on the contig containing the plasmid replicon IncHI2 (contig 15). In contrast, the MinION assembly (as-6) could attribute each resistance gene to the correct location (contig resembling plasmid or chromosome). While this assembly showed lower identity percentages compared to the MiSeq assembly, this did not result in incorrect AMR gene prediction. In the hybrid assembly, it was possible to correctly predict each gene with high accuracy and located on the correct contigs. This corresponds with the AMR gene prediction accuracies found in the MiSeq, MinION and hybrid assemblies of the MagCore, G500, phenol and phenol-ampli DNA extractions (Tables S6–S9). The number of point mutations detected with PointFinder differed between the assemblies (Table 3). The higher number of point mutations in the MinION assembly is likely caused by the higher error rate of the long-read sequencing. However, all point mutations had an unknown effect on resistance.

### 2.2. Further Optimization of the Workflow Using Flongle Flow Cells

For further optimization of the cost-efficiency of the workflow, the possibility of using the Flongle flow cells from Nanopore was explored. These flow cells have 1/10 of the pores of a MinION flow cell as capacity and would avoid the need for barcoding and hence waiting for a sufficient number of samples for a cost-efficient analysis. Furthermore, some parameters of the long-read library preparation were tested, i.e., shearing the DNA sample or using the long fragment buffer (LFB). The LFB can be used during the last cleaning step of the library preparation to remove short DNA fragments (<3000 bp) instead of the short fragment buffer (SFB) which enriches DNA of all fragment sizes. *E. coli* isolate COL20160015 was selected as a test case, as it was found to contain a large and low-copy *mcr-1*-containing plasmid, several smaller plasmids in higher copy numbers and a phage. The aim was to test if the Flongle produced enough reads to reconstruct all seven structures. As the genomic DNA extracts were found to be the most suited for plasmid reconstruction, G100 and MagCore DNA extracts were tested.

In Table 5, the Flongle read statistics, hybrid and Nanopore-only assemblies are shown. Compared to the assemblies made with the reads from the MinION run of *E. coli* COL20160015 (Figure 2 and Figure 3 and Figures S2 and S3 and Table 2 and Tables S10–S14), the Flongle produced comparable results. A hybrid assembly with Flongle data from *E. coli* COL20160015 (Flongles 1–4) was able to completely reconstruct both the chromosome and all of the seven plasmids. With hybrid assemblies of *E. coli* COL20160015 extracted with G100, the Flongle assembly (Flongles 1–3) performed better by resolving the 20 kb contig of a repetitive region in the chromosome that was seen in the hybrid assembly with MinION reads (Figure 2c). Surprisingly, this improvement was also seen in the Flongle run with fragmenting (Flongle 1), which was expected to be more comparable to the MinION run because the same Nanopore library preparation was used (1D ligation with Covaris g-TUBE shearing). The hybrid assembly obtained for the *E. coli* COL20160015 isolate extracted with MagCore (Flongle 4) performed slightly worse than the one obtained for that isolate extracted with Gtip 100 (Flongles 1–3), producing shorter repetitive regions in the chromosome (accession number: PRJNA646605).

While the assembly results of the Flongle with and without a fragmentation step are very similar (Flongles 1–4), the Flongles without fragmentation (Flongles 2–4) had a higher N50 but slightly lower output (total bases) in the Nanopore read statistics. The N50, output and median/average read quality of the Flongle without fragmenting and inclusion of the LFB (Flongle 3) was lower than that of the Flongle without fragmenting that used the short fragment buffer (SFB) (Flongles 2 and 4). This was in contrast to the expectations as LFB should have filtered out fragments smaller than 3 kb. Therefore, the library preparation protocol without fragmentation and with SFB (corresponding to Flongle 4) were applied to the MagCore DNA extraction of isolate *Salmonella* S15BD05371 to determine if the results were consistent in another bacterial species. Again, compared to the assemblies made with the reads from the MinION run of S15BD05371 (Figure 2 and Figure 3 and Figures S2 and S3 and Table 2 and Tables S10–S14), the Flongle produced comparable results (Table 5; Flongle 5). A hybrid assembly with data from Flongle 5 produced a more contiguous chromosome than with the MinION flow cell, but the plasmid was exactly the same. The AMR accuracy of all hybrid assemblies from Flongle reads corresponded with the AMR genes that were detected with the hybrid assemblies from MinION reads (Table 3, Tables S1 and S10–S14).

### 2.3. Application of the Workflow on Salmonella Kentucky Case Study

The developed workflow was subsequently applied to a case study of *Salmonella* Kentucky, for which the emergence in Europe of multidrug-resistant sequence type (ST) 198 isolates with a new type of ESBL gene (*bla*_CTX-M-14b_) has been reported [61]. The Belgian *Salmonella* National Reference Centre had determined via short-read sequencing that a large proportion of these Belgian *S.* Kentucky isolates were ESBL-positive. However, it was unknown whether the ESBL genes were localized on the chromosome or on a plasmid. Therefore, additional long-read sequencing was performed using MagCore DNA extracts from *S.* Kentucky isolated in Belgium (isolates S16BD08730, S18BD00684, S18BD03994 and S18BD05011).

The chromosomes of each isolate were assembled in one contig, and each isolate also showed one or multiple plasmids of which most were reconstructed in one contig (Figure S4). One plasmid from isolate S18BD00694 was split in multiple contigs, i.e., it was not possible to bridge a repetitive region in this plasmid due to the lower average read size in the MagCore DNA extracts. All isolates were determined to be of ST 198.

**Figure 5:**
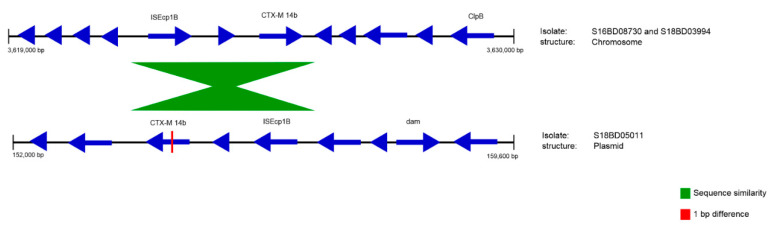
Similarity between the chromosomes of S16BD08730 and S18BD03394 and the plasmid of S18BD05011 (contig 2). The location and orientation of the genes is indicated with blue arrows. There is a 1 bp difference between the chromosomal and plasmid-located *bla*_CTX-M14b_ gene.

Table 6 shows the resistance genes that were found in the four isolates. The chromosomes of isolates S16BD08730, S18BD03394 and S18BD05011 share the AMR genes *aac(3)-Id, aph(3′′)-Ib, aph(3′)-Ia, aph(6)-Id, aadA7*, *sul1*, *tet(A)* and *aac(6′)-Iaa.* All these AMR genes except for *aac(6′)-Iaa* were localized very close to each other; by aligning this region to the NCBI nucleotide database, it was determined that these genes were part of a *Salmonella* genetic island 1 K (SGI1-K) [62,63]. Two isolates (S16BD08730 and S18BD03394) carried the ESBL gene *bla*_CTX-M-14b_ in the chromosome, while one isolate (S18BD00684) contained another ESBL gene, *bla*_TEM-1B_*,* in the chromosome. Moreover, the latter isolate also contained the ESBL gene *bla*_CMY-2_ on a plasmid. Isolate S18BD05011 contained no ESBL genes on its chromosome, but *bla*_CTX-M-14b_ and *bla*_TEM-1B_ were localized on two different plasmids, contigs 2 and 3, respectively. Each of these plasmids carried some other AMR genes as well (Table 6). ResFinder assigned *bla*_CTX-M-14b_ to isolate S18BD05011 with an identity of 99.89%, with a nonsynonymous point mutation at position 824. Upon further inspection, a region of 2850 bp including the ESBL gene was found to be similar in the chromosomes of S16BD08730 and S18BD03394 and in the plasmid (contig 2) of S18BD05011. In these regions, there was only 1 bp difference in the chromosomal and plasmid-located *bla*_CTX-M-14b_ gene. The ISEcp1B transposase, which is part of the IS1380 family, was detected in this region adjacent to the ESBL gene (Figure 5). In the NCBI database, there were no exact matches, but with a literature search, a description of this 2850 bp fragment was found in Lei et al. (2020) [64] in the chromosome of an *S.* Kentucky isolated from Chinese poultry.

While the *bla*_TEM-1B_ gene was also found in a chromosome (isolate S18BD00684) and a plasmid (contig 3 of isolate S18BD05011) of different isolates, the flanking regions were not similar. Therefore, it is unlikely that they are related to each other.

## 3. Discussion

AMR constitutes a huge public health concern. As plasmids contribute to the spread of AMR genes, their occurrence should be monitored. NGS is a powerful tool for the surveillance of AMR. However, it is difficult to reconstruct plasmids based on the commonly used short-read NGS because of the repetitive structures in plasmids. Therefore, it is challenging to determine whether AMR genes are located on the chromosome or on a plasmid within an isolate, which also hinders proper risk assessment and monitoring of circulating plasmids. In the current study, a strategy to reconstruct plasmids using NGS data in the context of AMR gene surveillance was determined. For this, several parameters at different levels of the workflow in both the wet and dry lab were evaluated (Figure 1). Within the workflow, the most optimal starting isolate (parameter 1), DNA extraction (parameter 2), sequencing technology (parameter 3) and bioinformatic analyses (parameter 4) were identified, using *mcr-1*-containing plasmids as case studies.

In a first attempt to reduce the complexity of plasmid reconstruction based on NGS data, a conjugation was performed in which a plasmid with unknown sequence was transferred to a lab host for which the genome sequence was known. In theory, this would facilitate the computational analyses, as the chromosomal reads could be filtered out in silico by mapping to the known genome sequence of the host strain, leaving only the plasmid reads to be used in assembly and hence for plasmid reconstruction. With only MiSeq reads, this filtering strategy did provide the AMR profile of the plasmid; however, the assembly was still very fragmented. Nevertheless, it was less fragmented than the MiSeq assembly without any chromosomal filtering step. With only MinION reads, the general structure of the plasmid could be retrieved; however, the size was incorrect, AMR genes had lower sequence identity to the ResFinder database than the MiSeq and hybrid assemblies and it was not possible to circularize the contig. A combination of the filtered MiSeq and MinION reads resulted in an almost complete reconstruction. However, it was missing a small fragment of ~1000 bp that it shared with the chromosome of the lab *E. coli*, and therefore any plasmid reads that contained this sequence were filtered out. The hybrid assembly of the conjugated sample without any filtering resulted in a complete chromosome and plasmid. Nevertheless, with whole-genome sequencing of conjugated isolates, a large part of the sequencing capacity is wasted, as the chromosomal sequence is already known. Moreover, as described in this study, it is possible for isolates to contain multiple plasmids, which can cause problems with the conjugation. Some plasmids will be nonconjugative (i.e., unable to transfer to another isolate or species) [2], or there will not be enough selection pressure if only antibiotics are administered of which the AMR genes are localized on other plasmids. However, as shown in this study, these nonconjugative plasmids can contain clinically relevant AMR genes, and these are unable to be characterized in the conjugated samples. Alternatively, transformation/electroporation could be considered. On the other hand, by transferring only one plasmid, it allows for the phenotypic effects to be studied with less interference of other genes, which would allow showing that the observed phenotype is the sole consequence of the presence of that particular plasmid. Because of the disadvantages combined with the fact that conjugation experiments take more hands-on time, it was decided that for the reconstruction of plasmids with hybrid assemblies it is more beneficial to start with the original isolate. However, it was seen that with the MiSeq reads of the conjugated samples where the chromosomal sequences were filtered out, it was possible to determine whether an AMR gene was located on the chromosome or a plasmid without full plasmid reconstruction. Therefore, if no long-read sequencing can be performed, it can be of added value to analyze conjugated samples with short-read sequencing only to determine the AMR profile of the transferred plasmid.

An alternative approach to reduce the chromosomal reads in the final output is to limit the input for the NGS analysis to plasmid DNA by adapting the DNA extraction protocol. As the goal is to use the workflow within a surveillance system in place in a National Reference Centre, it is important that the extraction method is cost-efficient with a limited hands-on time. Not all extraction methods are equally suited for the recovery of (large) plasmids. Moreover, long-read sequencing requires high-quality DNA, in large quantities and of high molecular weight [37]. Taking these restrictions into account, several plasmid extraction protocols were evaluated and compared to two whole-genome DNA extraction methods. This comparison was based on the quality parameters of the DNA and reconstruction of the plasmid with short (MiSeq) and long (MinION) sequencing reads and a combination of both. The plasmid DNA extractions were of much lower concentration than the whole-genome DNA extractions, even if an amplification step was performed. In the assemblies, plasmid DNA extractions contained many small incomplete contigs; without prior knowledge about the plasmid profile of the isolate, it would be difficult to determine whether these are of chromosomal or plasmid origin. Cloning vectors and naturally occurring plasmids can contain regions originating from chromosomes [65] or chromosomes can integrate plasmid sequences by homologous recombination [66], and therefore it becomes difficult to determine if these unexpected linear contigs are part of chromosomes or plasmids. The only plasmid DNA extraction which did not contain incomplete contigs was the phenol chloroform combined with an exonuclease and amplification of the DNA. However, the lower concentration still caused problems in the MinION sequencing, making the hybrid assembly of lower quality than the whole-genome DNA extractions. Moreover, this plasmid extraction protocol is more laborious and expensive than the whole-genome extraction. Although the proportion of reads mapping to the plasmid was much lower in whole-genome extracts (~5% vs. >50%), these reads were still sufficient for fully reconstructing the plasmid. Furthermore, with the whole-genome DNA extractions, the copy number of the plasmid could also be estimated by comparing the plasmid coverage to that of the chromosome. Although the tested plasmid DNA extractions were able to reconstruct the large, low-copy *mcr-1* plasmid, the whole-genome DNA extractions performed equally as well or better in all tested parameters. Therefore, the whole-genome DNA extractions were chosen for the workflow. However, if only short-read sequencing can be performed and it concerns a crucial isolate for which a specific, more time-consuming DNA extraction protocol can be followed, then the phenol chloroform plasmid DNA extraction with exonuclease and amplification can be useful to determine the plasmid content.

The most reliable reconstruction of plasmids was with hybrid assemblies from the whole-genome Genomic Tip 100 and MagCore DNA extracts of the original isolate. A benefit for AMR surveillance of reconstructing plasmids with whole-genome assemblies is that simultaneously chromosomal and plasmid AMR genes can be determined. While the Genomic Tip 100 is more expensive and more time-consuming per sample than the automated MagCore DNA extractions, it also produced more consistently longer Nanopore reads. However, the smaller MinION reads from the MagCore extract were still sufficient to cover all repetitive regions in the hybrid assembly of the used case study. Therefore, both (or similar whole-genome DNA extractions) can be used in the workflow. However, even though the MagCore showed lower DNA purities and inconsistent fragment sizes, it is more adapted to a routine surveillance setting than the Genomic Tip 100 extractions as the MagCore extractions are more cost-efficient and require less hands-on time (semiautomatization). Based on the DNA quality tests, it seemed that concentration and fragment sizes had a larger effect on the Nanopore read length and yield than the purity of the DNA extract. The contaminants that caused the lower purity in the MagCore extracts are likely removed during the cleaning steps with the magnetic AMPure XP beads in the library preparation of the 1D ligation sequencing kit.

In this study, Unicycler was used for all assemblies to keep the workflow consistent. Unicycler is a pipeline consisting of several tools that perform the assembly, error correction and circularization of contigs. It has been shown to produce very accurate hybrid assemblies [67]. Another advantage of Unicycler is that it is relatively user-friendly, requiring a single command on the command line. Although Unicycler was used in a Linux environment, which might be more difficult to put into place in a routine surveillance setting, there are currently no hybrid assemblers that are usable in Windows. However, a user-friendly GUI is available for nonexperts [68]. Nevertheless, there are other (hybrid) assemblers available that specifically focus on plasmids (e.g., plasmidSPAdes) [69] or do reference-guided assemblies (e.g., PLACNETw) [70] or that focus just like Unicycler on the whole genome including plasmids (e.g., Canu) [71]. However, plasmidSPAdes has been shown to have difficulties with assembling large and low-copy plasmids [27]. Furthermore, reference-guided assemblers such as PLACNETw could misassemble plasmids that contain rearrangements and plasmids that are not present in the database or of which there are only low-quality references in the database. It would be interesting to make an in-depth comparison between all available software in a dedicated future study.

Based on the above, it was seen that a hybrid assembly from a whole-genome extraction of the original isolate was the best approach for accurate plasmid reconstruction using NGS data. Next, it was determined which sequencing technology and assembly method resulted in the most accurate AMR gene prediction and localization. The similarity of the detected genes to the ones in the AMR databases used (percent identity) was higher in MiSeq assemblies than in the Nanopore assemblies, which can be explained by the lower error rate of short-read sequencing. With the lower similarity, it was, however, still possible to determine the correct variant in the Nanopore assemblies. Nevertheless, care should be taken, as for gene variants that only differ by a few base pairs (for example *bla*_CTX-M_), this could result in the wrong gene prediction. Moreover, the detection of the correct gene or variant is also dependent on the content of the database and alignment software. It would be helpful for AMR gene databases to be harmonized and kept continuously updated in the future. Furthermore, the Nanopore assembly is not suitable for the prediction of chromosomal mutations (SNPs) that confer AMR. As to the localization of the AMR genes, in MiSeq assemblies it was impossible to determine whether the AMR gene was located on a chromosome or plasmid, in contrast to the MinION assemblies. Therefore, hybrid assemblies combined the best of both worlds, i.e., resulting in both very accurate AMR gene prediction and correct localization, making them the most suitable for the workflow.

As hybrid assemblies gave the most complete assemblies of plasmids with accurate AMR gene prediction, both short and long sequencing reads would be necessary. In this study, Nanopore sequencing was chosen, as this portable technology is accessible to more labs and has a lower initial investment cost compared to PacBio. Nanopore technologies offer two types of flow cells, i.e., the already widely used MinION and the newer Flongle. Although the Flongle is cheaper, it has a lower pore count and therefore produces less output. However, this makes this flow cell interesting for analyzing a single sample at a time, thereby avoiding the need for barcoding of samples when using the MinION flow cell, which requires the grouping of ~12 samples to be cost-effective. While the MinION can be used to save time if multiple samples need to be analyzed simultaneously, between 1–5% of the reads are lost because the barcode cannot be recognized. Another disadvantage of barcoding is the occurrence of cross-over contamination between barcodes, which is mostly caused by chimeric reads [72]. While this contamination has been shown to not affect hybrid assemblies [57], it could result in erroneous alignments when the sequencing reads are used for this purpose. The use of a Flongle will avoid this issue and will also allow for a more rapid turn-around time of a hybrid assembly analysis in a daily routine setting, as there is no need to wait until a sufficient number of samples are collected to start a MinION analysis. Additionally, the Flongle requires less input DNA per sample, i.e., 500 ng compared to 1 µg for MinION flow cells. For these reasons, we compared the sequencing output and hybrid assemblies from the MinION to the Flongle and determined that the Flongle output is more than sufficient to reconstruct plasmids and also the bacterial chromosome. Moreover, the AMR prediction accuracy of the hybrid and Nanopore-only assemblies from Flongle data was similar to that of assemblies with MinION sequencing reads. Therefore, the Flongle seems more suitable for the workflow, except when a lot of isolates need to be sequenced in parallel.

One issue with Nanopore sequencing, in contrast to the routinely used short-read sequencing technology, is that the protocols for library preparation as recommended by Oxford Nanopore Technologies are constantly evolving. When starting this study, it was recommended to fragment the input DNA to 8 kb during the library preparation with the 1D ligation sequencing kit SQK-LSK108 to increase the sequencing output. Since the introduction of the ligation sequencing SQK-LSK109 kit, shearing of input DNA was only recommended when using amounts of DNA which are below the specifications of Oxford Nanopore Technologies. Moreover, the 1D ligation sequencing kit was chosen for its higher sequencing output and reliability at the start of the study. Another library preparation kit that has been used on plasmids is the rapid sequencing kit (SQK-RAD). With the rapid sequencing kit, less manipulations are required in the library preparation, which may be useful in obtaining longer reads or even ultralong reads (>100 kb) [33,73,74]. However, the rapid sequencing kit does not have any cleaning steps during the library preparation, and therefore a larger effect of the purity of the DNA on the sequencing results might be seen than with the 1D ligation sequencing kit. In this study, different library preparations were compared to determine the effect of DNA fragmentation on sequencing output, read length and plasmid reconstruction. The results showed that fragmentation increased the obtained sequencing output; however, this was at the expense of the average read size, which was smaller compared to that obtained with unfragmented samples. However, both with and without fragmentation, the plasmid was completely reconstructed with high accuracy for all expected AMR genes. While the read size did not matter for the assembly of our specific case study, it is advisable not to shear the DNA of the isolate if the DNA concentration is within the recommendations of Oxford Nanopore Technologies (>500 ng for Flongle and >1000 ng for MinION) to ensure that all repetitive regions can be bridged.

Performing both short- and long-read sequencing for each isolate can be costly, especially in a surveillance context where many isolates need to be analyzed. Therefore, it would be recommended to first perform short-read sequencing on all isolates to determine if there are indications of unique or otherwise relevant plasmids or clinically relevant AMR genes, and then select those for additional long-read sequencing. While with MiSeq sequencing only it was not possible to accurately reconstruct plasmids, it is possible to get an indication as to whether there were sequences that likely come from plasmids using databases like PlasmidFinder [52] and PLSDB [53] or machine-learning tools [75]. However, there are still improvements possible in these databases and tools as, for example, some plasmids contain multiple or zero of the replicons described in the PlasmidFinder database [52,76], and machine learning is highly dependent on the training dataset and comes with its own biases and pitfalls [77,78,79]. In the future, it is likely that the accuracy and output of long-read sequencing keeps improving, and thus it would then be possible to replace hybrid assemblies with assemblies made solely from long-read sequences if accurate AMR gene detection and localization is of interest. The R10 flow cells are reported to have higher accuracy. It would be interesting to test whether the accuracy is sufficient to be able to reduce our workflow to long-read sequencing only.

Our optimal workflow for plasmid reconstruction, consisting of whole-genome DNA extraction on the original isolate followed by MiSeq and Nanopore sequencing to perform hybrid assemblies, was established using isolates with a large *mcr-1*-containing plasmid. Without application of this workflow, it would not have been possible to reconstruct this large, low-copy plasmid of >200 kb. Besides the *mcr-1* gene, the plasmid contained ESBL genes and other critically important AMR genes [80]. We applied the workflow both to *E. coli* and *Salmonella*. The NCBI nucleotide database contained sequences similar to that of the *mcr-1* plasmid from the *E. coli* isolates (COL20160015 and R274) but did not contain a sequence that completely covered the *mcr-1* plasmid from the *Salmonella* isolate (S15BD05371). This highlights that the public databases are currently lacking sequences of circulating plasmids. To be able to trace these plasmids and prevent their spread, a more complete characterization of plasmids and submission of these sequences to public repositories are needed.

Finally, our workflow was applied to an ESBL-positive *Salmonella* Kentucky case study. ESBL-positive *S.* Kentucky are part of the list of multidrug-resistant pathogens that have been designated as a high-priority issue by the WHO [81]. In the beginning of the 21st century, European ST198 *Salmonella* Kentucky were susceptible to antibiotics; however, there was a rapid increase of mutations in the *parC* and *gyrA* genes that are involved in ciprofloxacin resistance. Of extra concern is the recent acquisition of bla_CTX-M-14b_ gene reported in ST198 *S.* Kentucky that was not seen before and had already started to spread throughout Europe [61,82]. At the start of this study, it was unknown whether the ESBL AMR genes detected by short-read sequencing were localized on a plasmid or on the chromosome. Without this knowledge, it is more difficult to assess the risk of spread of this AMR, because while chromosomal ESBL genes spread clonally within the *S.* Kentucky population, it has the potential to spread to other bacterial pathogens if it is localized on a plasmid. With the hybrid assemblies obtained with the workflow, it was determined that in the Belgian *S.* Kentucky isolates, the ESBL genes were present on both the chromosome and on plasmids and that there was also a likely exchange by a transposase of a 2850 bp region between the chromosome and a plasmid of different isolates. The results of our workflow on the Belgian isolates, and especially of the plasmid reconstruction, were put into epidemiological context of the other European *Salmonella* Kentucky ESBL-positive isolates by Coipan et al. [83]. They found that this 2850 bp fragment was present in the chromosome and plasmids of other *S.* Kentucky isolates [83]. The exchanged fragment is very similar to that on the chromosome of an *S.* Kentucky isolated from poultry in China [64]; however, the exact sequence reported in the Chinese study is not yet available in public databases to make the full comparison. Other surveillance and in vitro studies have also shown the role of this transposase in transfer of *bla*_CTX-M_ genes from plasmids to chromosomes of bacteria [84]. Due to the lack of data in current databases, it could not be determined whether the 2850 bp fragment was first localized on the chromosome and then transferred to the detected plasmid or vice versa. However, the presence of the ESBL gene on the plasmid increases its transmissibility, which is a public health risk. Without the application of our developed workflow, this knowledge would not have been available.

This study describes a systematic evaluation of wet and dry lab components involved in accurate reconstruction of plasmids and characterization of their AMR gene content based on NGS data. Our developed workflow will contribute to an improved monitoring of circulating plasmids and assessment of their transfer risk.

## 4. Material and Methods

### 4.1. Bacterial Isolates

The bacterial strains used in this study were pathogenic *E. coli* (Col20160015), *Salmonella enterica* subsp. *enterica* serovar Typhimurium (S15BD05371) and serovar Kentucky (S16BD08730, S18BD00684, S18BD03994, S18BD05011) isolated from human clinical samples. The plasmid of Col20160015 was transferred to a known bacterial host (*E. coli* C600 from BCCM) by conjugation (R274). Table 1 describes all DNA extractions performed on these isolates, and Table 7 describes the known characteristics of these isolates.

All isolates were grown on LB plates overnight at 37 °C, and then one colony was transferred to LB broth of varying volumes depending on the extraction protocol (see Section 4.4).

### 4.2. Antimicrobial Susceptibility

Antibiotic susceptibility was assessed using broth microdilution, with the commercially available Sensititre EU Surveillance *Salmonella*/*E. coli* EUVSEC Plate (Thermo Fisher Scientific, Waltham, MA, USA) according to the manufacturer’s instructions.

### 4.3. Conjugation

The donor strain, pathogenic *E. coli*, was transferred to Mueller–Hinton (MH) agar plates with colistin (1 µg/mL). After overnight incubation at 37 °C, one colony was transferred to 2 mL MH medium with colistin (1 µg/mL). Simultaneously, the receptor strain (*E. coli* C600 from BCCM) was cultured in MH medium with rifampicin (50 µg/mL) to induce spontaneous mutations in the *rpoB* gene that confer resistance to rifampicin. Then, the plasmid was transferred by mixing the donor and receptor strain according to Makart et al. [85]. This mix was incubated for 24 h at 37 °C. Subsequently, it was centrifuged for 5 min at 8000× *g*. The supernatant was removed and the pellet resuspended in 100 µL demineralized sterile water. Then, 10 µL of the resuspension was transferred to a colistin/rifampicin (1 µg/mL and 50 µg/mL) MH agar plate. This plate was incubated overnight at 37 °C.

The next day, colonies were counted to determine the transfer efficiency. Then, one colony was transferred to a new colistin/rifampicin MH agar plate to make a glycerol stock for further use and experiments.

### 4.4. DNA Extractions

For all DNA extractions, one colony was transferred to LB broth and then incubated overnight at 37 °C. For the isolates with the *mcr-1* plasmid, 1 µg/mL of colistin was supplied to the culture media.

#### 4.4.1. Whole-Genome DNA Extraction with Genomic Tip 100

The total genomic content (chromosome and plasmid) of isolates COL20160015, R274 and S15BD05371 was extracted from 10 mL of culture using the Genomic-tip 100/G according to the manufacturer’s instructions (Qiagen Benelux B.V., Venlo, The Netherlands).

#### 4.4.2. Whole-Genome DNA Extraction with MagCore Genomic DNA Bacterial Kit

The total genomic content (chromosome and plasmid) of isolates COL20160015, S15BD05371, S16BD08730, S18BD00684, S18BD03994 and S18BD05011 was extracted from 10 mL of culture using MagCore Genomic DNA Bacterial Kit according to the manufacturer’s instructions (RBCBioscience, New Taipei City, Taiwan). All samples were extracted on the same cartridge.

#### 4.4.3. Plasmid Extraction with Genomic Tip 500 and Plasmid Extraction Buffers

The plasmid of isolate S15BD05371 was extracted from 500 mL of culture using the Genomic-tip 500/G and plasmid extraction buffers according to the manufacturer’s instructions (Qiagen Benelux B.V., Venlo, The Netherlands) described in the ‘Isolation of large-construct DNA using QIAGEN Plasmid Maxi kit’ protocol.

#### 4.4.4. Plasmid DNA Extraction Genomic Tip 500, Plasmid Extraction Buffers and an Exonuclease

The plasmid of isolate S15BD05371 was extracted from 500 mL of culture using the Genomic-tip 500/G, plasmid extraction buffers and an exonuclease step according to the manufacturer’s instructions (Qiagen Benelux B.V., Venlo, The Netherlands) as described in the ‘Isolation of genomic DNA-free DNA using the QIAGEN Large-Construct kit Plasmid’ protocol.

#### 4.4.5. Plasmid DNA Extraction with Phenol Chloroform

The plasmid of isolate S15BD05371 was extracted from 5 mL of culture based on the phenol chloroform extraction described in Hoffmann et al. (2017) [86].

#### 4.4.6. Plasmid DNA Extraction with Phenol Chloroform, an Exonuclease and Amplification

The same DNA extraction as in Section 4.4.5 was performed, but the extract was treated with Plasmid-Safe ATP-dependent exonuclease, incubated at 37 °C for 30 min and then inactivated at 70 °C for 30 min. This was followed by an ethanol precipitation clean-up. Then, 5 µL of the DNA extract was amplified at 30 °C for 12 h with the Qiagen REPLI-g Mini kit.

### 4.5. Quality Control of DNA

A Nanodrop 2000 (Thermo Fisher Scientific Waltham, MA, USA) device was used to determine the purity of the DNA. The concentration of the DNA was determined with a Qubit 3.0 fluorometer (Thermo Fisher Scientific, Waltham, MA, USA). The Agilent 4200 Tapestation (Agilent Technologies, Santa Clara, CA, USA) was used to determine the fragment length of the DNA and the DNA integrity (DIN). The requirements as described by Oxford Nanopore for the DNA were 500 ng (Flongle) or 1 µg (MinION), A260/A280 = 1.8, A260/A230 = 2.0–2.2 and fragment sizes of >30 kb [37].

### 4.6. qPCR Reactions

The presence of the plasmids and chromosome in the DNA extract was determined with qPCR reactions (*mcr-1* and 16S) using previously described assays [87,88]. The qPCRs were performed in reactions with a final volume of 25 μL containing 1× SYBR Green Master Mix (Molzym), 250 nM of the forward and reverse primer and 10 ng of the extracted DNA. The MCR-1 qPCR cycle conditions consisted of a denaturation step at 95 °C for 2 min followed by 40 cycles of 3 s at 95 °C, 20 s at 60 °C and 7 s at 72 °C. The 16S qPCR cycle conditions consisted of a denaturation step at 95 °C for 10 min, followed by 40 cycles of 15 s at 95 °C and 1 min at 62 °C.

### 4.7. Next-Generation Sequencing

Short-read sequencing libraries were prepared with an Illumina Nextera XT DNA Library Preparation Kit and sequenced on an Illumina MiSeq instrument with a 250 bp paired-end protocol (MiSeq v3 chemistry) according to the manufacturer’s instructions. Trimming of the short reads was performed with Trimmomatic (version 0.32) [89]. First, the Illuminaclip option was used to remove the Nextera adapter sequences. Then, a sliding window approach of four bases and trimming when the Phred score dropped below 30 was employed. Lastly, the leading and trailing bases of a read were removed when the Phred score dropped below 3. All reads that were smaller than 50 bp were removed.

The MinION long-read sequencing library was prepared by using the 1D ligation sequencing kit (SQK-LSK108, Oxford Nanopore) according to the manufacturer’s protocol for genomic DNA without barcoding. In total two MinION flow cells were used, and libraries with 8 and 12 barcodes were loaded on them respectively (EXP-NBD103, Oxford Nanopore). From each isolate, 1 µg of DNA was used at the start of the protocol. The optional step of shearing the DNA to 8 kb fragments with Covaris G tubes was included, while repairing the DNA was omitted. The sequencing was carried out on a R9.4 flow cell (Oxford Nanopore) and sequenced for 48 h.

For the Flongle long-read sequencing libraries, the adapted 1D ligation protocol for Flongles was used with the SQK-LSK109 sequencing kit. From each isolate, 500 ng of DNA was used at the start of the protocol. DNA repair was no longer optional in SQK-LSK109, and therefore this was performed for the Flongle runs. In the SQK-LSK109, there are two washing buffers, the SFB and LFB, of which the latter enriches for DNA fragments >3000 bp. Both these washing buffers and the inclusion or exclusion of the shearing step were used on separate Flongle flow cells. Moreover, no barcoding was performed on the Flongles.

Basecalling and demultiplexing of the Nanopore sequences was performed with Guppy (3.2.4). Then, all Nanopore reads with a quality score lower than 7 or a length lower than 1000 bp were removed with NanoFilt [90]. For the output of the sequencing runs and the theoretical coverage of each sample, see Table S15. The statistics of the Illumina reads were determined with FastQC [91], and those of the Nanopore reads were determined with NanoStat [90]. Raw sequencing data and the de novo assemblies were submitted to NCBI Sequence Read Archive (SRA) [92] and NCBI GenBank [93] under the accession numbers PRJNA646605 and PRJNA641774.

### 4.8. Bioinformatics Analysis

De novo hybrid assembly with Nanopore and Illumina reads was carried out using Unicycler (version 0.4.8) at default parameters [67]. The following tools were used in the Unicycler pipeline: SPAdes (version 3.13.0), Miniasm (version 0.3), Racon (version 1.4.3), makeblastdb (version 2.7.1+), tblastn (version 2.7.1+), Bowtie2 (version 2.3.4.3), Samtools (version 1.9), Java (version 10.0.1) and Pilon (1.23) [94,95,96,97,98,99,100]. Visualization and finishing of the Unicycler assembly were performed in Bandage (version 0.8.1) [101]. ResFinder (version 3.2) [52] was used to determine genes responsible for the genotypic AMR resistance. PlasmidFinder (version 2.1) [52] was used for the detection of plasmid replicons. PointFinder (version 3.1) [102] was used for the detection of chromosomal mutations that confer antibiotic resistance. The presence of the phage sequence was confirmed with PHASTER [103]. BWA-MEM (version 0.7.12-r1039) was used for the mapping of MiSeq reads [104]. Minimap2 (version 2.14) was used for mapping the Nanopore reads [105]. Assembly statistics were retrieved with Quast (version 5.0.2) and BBmap (version 38.33) [106,107].

## Figures and Tables

**Table 1:** DNA extraction methods applied in this study, their mechanism and isolates used.

Code	Isolate	Species	Extraction Method	Content Extracted	Mechanism Extraction
extr-1	COL20160015	*E. coli*	G100	whole genome	anion-exchange column
extr-2	R274	*E. coli*	G100	whole genome	anion-exchange column
extr-3	S15BD05371	*Salmonella*	G100	whole genome	anion-exchange column
extr-4	S15BD05371	*Salmonella*	MagCore	whole genome	semiautomatic DNA extraction based on magnetic beads
extr-5	S15BD05371	*Salmonella*	G500	plasmid	denaturation chromosome and then separation with anion-exchange column
extr-6	S15BD05371	*Salmonella*	G500-exo	plasmid	denaturation chromosome and then separation with anion-exchange column ^1^
extr-7	S15BD05371	*Salmonella*	phenol	plasmid	denaturation chromosome and then separation with phenol chloroform
extr-8	S15BD05371	*Salmonella*	phenol-ampli	plasmid	denaturation chromosome and then separation with phenol chloroform ^1, 2^
extr-9	COL20160015	*E. coli*	MagCore	whole genome	semiautomatic robot DNA extraction based on magnetic beads
extr-10	S16BD08730	*Salmonella* Kentucky	MagCore	whole genome	semiautomatic robot DNA extraction based on magnetic beads
extr-11	S18BD00684	*Salmonella* Kentucky	MagCore	whole genome	semiautomatic robot DNA extraction based on magnetic beads
extr-12	S18BD03994	*Salmonella* Kentucky	MagCore	whole genome	semiautomatic robot DNA extraction based on magnetic beads
extr-13	S18BD05011	*Salmonella* Kentucky	MagCore	whole genome	semiautomatic robot DNA extraction based on magnetic beads

^1^ DNA extraction is followed up by treatment with an exonuclease to digest chromosomal DNA. ^2^ Exonuclease treatment is followed up by PCR amplification with a Phi 29 DNA polymerase.

**Table 2:** Assembly statistics of *Salmonella* isolate S15BD05371 with MiSeq and MinION reads and a combination (hybrid).

Code	Extraction Method	NGS Data Used	Number of Contigs	Length Total Assembly	Size of Top 5 Longest Contigs	Circular Contigs	Dead Ends	Connected Components
as-1	G100	MiSeq	105	5,177,228	contig 1 = 934,695	0	9	2
					contig 2 = 602,224			
					contig 3 = 376,547			
					contig 4 = 315,791			
					contig 5 = 282,528			
as-2	MagCore	MiSeq	106	5,178,799	contig 1 = 935,416	0	0	1
					contig 2 = 602,340			
					contig 3 = 329,197			
					contig 4 = 319,029			
					contig 5 = 315,791			
as-3	G500	MiSeq	403	5,085,365	contig 1 = 213,752	0	614	289
					contig 2 = 90,183			
					contig 3 = 87,956			
					contig 4 = 71,127			
					contig 5 = 70,825			
as-4	phenol	MiSeq	424	5,036,011	contig 1 = 218,263	0	754	358
					contig 2 = 89,895			
					contig 3 = 68,292			
					contig 4 = 62,358			
					contig 5 = 58,698			
as-5	phenol-ampli	MiSeq	4	223,724	contig 1 = 218,260	0	0	1
					contig 2 = 3241			
					contig 3 = 1451			
					contig 4 = 772			
as-6	G100	MinION	2	5,234,218	contig 1 = 5,009,299	contigs 1 and 2	0	2
					contig 2 = 224,919			
as-7	MagCore	MinION	2	5,235,338	contig 1 = 5,010,386	contigs 1 and 2	0	2
					contig 2 = 224,952			
as-8	G500	MinION	4	5,264,667	contig 1 = 3,271,687	contigs 3 and 4	4	4
					contig 2 = 1,766,994			
					contig 3 = 224,940			
					contig 4 = 1046			
as-9	phenol	MinION	12	237,905	contig 1 = 49,994	0	24	12
					contig 2 = 43,844			
					contig 3 = 27,329			
					contig 4 = 26,481			
					contig 5 = 26,154			
as-10	phenol-ampli	MinION	-	-	-	-	-	-
as-11	G100	Hybrid (MiSeq + MinION)	9	5,242,330	contig 1 = 4,924,297	contig 2	0	2
					contig 2 = 225,369			
					contig 3 = 87,966			
					contig 4 = 2562			
					contig 5 = 1569			
as-12	MagCore	Hybrid (MiSeq + MinION)	2	5,243,584	contig 1= 5,018,218	contigs 1 and 2	0	2
					contig 2 = 225,366			
as-13	G500	Hybrid (MiSeq + MinION)	5	5,248,759	contig 1 = 5,018,851	contig 2	8	5
					contig 2 = 225,365			
					contig 3 = 1904			
					contig 4 = 1333			
					contig 5 = 1306			
as-14	phenol	Hybrid (MiSeq + MinION)	67	5,112,587	contig 1 = 365,603	contig 5	94	41
					contig 2 = 307,717			
					contig 3 = 225,575			
					contig 4 = 224,243			
					contig 5 = 225,369			
as-15	phenol-ampli	Hybrid (MiSeq + MinION)	3	224,594	contig 1 = 222,371	0	0	1
					contig 2 = 1451			
					contig 3 = 772			

A contig is determined to be circular when it shows a connection in the assembly graph (Figure 3) from its own 5′ end to its 3′ end. A dead end is counted each time a side (5′ or 3′) of a contig has no connection to another contig or itself. A connected component is counted as multiple contigs that are connected to each other in the assembly graph. If a contig has no connections to any other contig (2 dead ends), it is also counted as one connected component.

**Table 3:** Antimicrobial resistance genes, plasmid replicons and chromosomal mutations detected with ResFinder, PlasmidFinder and PointFinder in whole-genome MiSeq, MinION and hybrid assembly of *Salmonella* isolate S15BD05371 extracted with the Genomic Tip 100.

**ResFinder**	**MiSeq**			**MinION**			**Hybrid**		
**Resistance Gene**	**Contig**	**Identity**	**Query/Template Length**	**Contig**	**Identity**	**Query/Template Length**	**Contig**	**Identity**	**Query/Template Length**
*aac(3)-IV* ^1^	14	100	777/777	**2**	**99.9**	**777/777**	**2**	**100**	**777/777**
*aac(6′)-Iaa* ^1^	1	100	438/438	1	100	438/438	1	100	438/438
*aadA2b* ^1^	40	99.9	780/780	**2**	**99.6**	**780/780**	**2**	**99.9**	**780/780**
*aph(4)-Ia* ^1^	14	100	1026/1026	**2**	**100**	**1026/1026**	**2**	**100**	**1026/1026**
*bla*_TEM-1B_^2^	14	100	861/861	**2**	**99.8**	**861/861**	**2**	**100**	**861/861**
*mcr-1.1* ^3^	**15**	**100**	**1626/1626**	**2**	**99.9**	**1626/1626**	**2**	**100**	**1626/1626**
*lnu(F)* ^4^	40	100	761/822	**2**	**99.6**	**761/822**	**2**	**100**	**761/822**
*qnrS1* ^5^	54	100	657/657	**2**	**99.9**	**657/657**	**2**	**100**	**657/657**
*sul3* ^6^	42	100	792/792	**2**	**99.9**	**793/792**	**2**	**100**	**792/792**
*tet(A)* ^7^	14	100	1200/1200	**2**	**99.7**	**1200/1200**	**2**	**100**	**1200/1200**
*tet(B)* ^7^	33	100	1206/1206	1	99.7	1207/1206	1	100	1206/1206
*dfrA12* ^8^	40	100	498/498	**2**	**99.8**	**498/498**	**2**	**100**	**498/498**
**PlasmidFinder**	**MiSeq**			**MinION**			**Hybrid**		
**Plasmid**	**Contig**	**Identity**	**Query/Template Length**	**Contig**	**Identity**	**Query/Template Length**	**Contig**	**Identity**	**Query/Template Length**
IncHI2	15	100	327/327	2	100	327/327	2	100	327/327
IncHI2A	15	99.5	630/630	2	99.1	630/630	2	99.5	630/630
**PointFinder**	**MiSeq**			**MinION**			**Hybrid**		
total amount of mutations	5			221			6		

In bold and underlined are the AMR genes of which it is certain that they are located on a plasmid. Query/template length indicates the coverage of the gene or replicon, and this is identical for the MiSeq, MinION and hybrid assembly. ^1^ Aminoglycoside; ^2^ beta-lactam; ^3^ colistin; ^4^ macrolide; ^5^ quinolone; ^6^ sulfonamide; ^7^ tetracycline; ^8^ trimethoprim.

**Table 4:** Minimum inhibitory concentration (MIC) of *Salmonella* isolate S15BD05371 and the responsible genes for the antibiotics where an MIC higher than the EUCAST cut-off was observed [60].

Antibiotic	EUCAST Cut-Off (mg/L)	MIC of S15BD05371 (mg/L)	Responsible Gene ^3^
Ampicillin	8	>64	*bla*_TEM-1B_
Azithromycin	16	4	-
Cefotaxime	2	≤0.25	-
Ceftazidime	4	≤0.5	-
Chloramphenicol	8 ^1^	16	-
Ciprofloxacin	0.5	0.5	-
Colistin	2	4	*mcr-1.1*
Gentamicin	2	4	*aac(3)-IV, aac(6′)-Iaa*
Meropenem	8	≤0.03	-
Nalidixic Acid	- ^2^	8	*qnrS1*
Sulfamethoxazole	- ^2^	>1024	*sul3*
Tetracycline	4	>64	*tet(A)* and *tet(B)*
Tigecycline	0.5	0.5	-
Trimethoprim	4	>32	*dfrA12*

All chromosomal point mutations had an unknown effect on the resistance profile in *Salmonella* isolate S15BD05371. ^1^ The ECOFF value for chloramphenicol in *Salmonella* is 16 mg/L. ^2^ There is no EUCAST cut-off MIC value available for these antibiotics. ^3^ The antibiotics to which *aadA2b*, *aph(4)-Ia* and *lnu(F)* confer resistance were not covered by the EUVSEC plate used for the standard surveillance MIC testing.

**Table 5:** Nanopore read and hybrid assembly statistics of Flongle runs (raw reads) with variations in the library preparation.

Isolate	*E. coli* COL20160015	*E. coli* COL20160015	*E. coli* COL20160015	*E. coli* COL20160015	*Salmonella* S15BD05371
DNA Extraction	G100	G100	G100	MagCore	MagCore
**Flongle Read Statistics**					
Variation Library prep	Fragmentation to 8 kb	No Fragmentation	No Fragmentation + Long Fragment Buffer	No Fragmentation	No Fragmentation
Washing buffer used	SFB	SFB	LFB	SFB	SFB
Flongle id	Flongle 1	Flongle 2	Flongle 3	Flongle 4	Flongle 5
Mean read length	7368	6459	4334	7189	7200
Mean read quality	7.5	7.4	6.2	7.2	7.8
Median read length	7118	1757	1343	3483	4202
Median read quality	7.8	7.7	6.2	7.6	8
Number of reads	117,788	107,610	114,758	100,353	182,252
Read length N50	11,059	21,861	14,190	15,787	13,950.00
Total bases	867,857,659	695,075,675	497,339,098	721,405,028	1312,154,207
**Hybrid assembly statistics**					
size contig 1 (bp)	5,345,374	5,345,375	5,345,375	5,343,055	5,018,079
size contig 2 (bp)	238,073	238,073	238,073	238,070	225,369
size contig 3 (bp)	108,394	108,394	108,394	108,394	
size contig 4 (bp)	87,936	87,936	87,936	87,862	
size contig 5 (bp)	41,216	41,216	41,216	41,195	
size contig 6 (bp)	11,988	11,988	11,988	11,988	
size contig 7 (bp)	3904	3904	3904	3904	
**Nanopore-only assembly statistics**					
size contig 1 (bp)	4,047,777	5,394,891	5,362,109	5,411,488	5,007,766
size contig 2 (bp)	1,348,910	237,434	237,000	237,580	224,949
size contig 3 (bp)	237,636	107,955	113,757	135,019	
size contig 4 (bp)	108,172	87,986	107,875	118,065	
size contig 5 (bp)	88,111	74,976	87,901	70,504	
size contig 6 (bp)	66,700	65,323	11,911	66,926	
size contig 7 (bp)	37,157	41,367		57,461	
size contig 8 (bp)	11,950	41,950		46,472	
size contig 9 (bp)		18,467		46,038	
size contig 10 (bp)		11,927		42,412	
size contig 11 (bp)		3889		40,692	
size contig 12 (bp)				40,556	
size contig 13 (bp)				11,977	
size contig 14 (bp)				3892	

SFB = short fragment buffer; LFB = long fragment buffer.

**Table 6:** ResFinder and PlasmidFinder output of the *Salmonella* Kentucky isolates S16BD08730, S18BD00684, S18BD03994 and S18BD05011.

**ResFinder**	**S16BD08730**			**S18BD03394**			**S18BD05011**			**S18BD00684**		
**Resistance Gene**	**Contig**	**Identity**	**Query/Template Length**	**Contig**	**Identity**	**Query/Template Length**	**Contig**	**Identity**	**Query/Template Length**	**Contig**	**Identity**	**Query/Template Length**
*aac(3)-IIa* ^1^	-	-	-	-	-	-	**3**	**100**	**861/861**	-	-	-
*aac(3)-Id* ^1^	1	100	477/477	1	100	477/477	1	100	477/477	-	-	-
*aac(6* *′)-Iaa* ^1^	1	98.63	438/438	1	98.63	438/438	1	98.63	438/438	1	98.63	438/438
*aadA1* ^1^	-	-	-	-	-	-	**2**	**100**	**789/789**	-	-	-
*aadA7* ^1^	1	100	798/798	1	100	798/798	1	100	798/798	-	-	-
*aph(3* *′′)-Ib* ^1^	1	100	804/804	1	100	804/804	1	100	804/804	-	-	-
*aph(3* *′)-Ia* ^1^	1	100	816/816	1	100	816/816	1	100	816/816	-	-	-
*aph(6)-Id* ^1^	1	100	837/837	1	100	837/837	1	100	837/837	-	-	-
*bla*_CMY-2_^2^	-	-	-	-	-	-	-	-	-	**2**	**100**	**1146/1146**
*bla*_CTX-M-14b_^2^	1	100	876/876	1	100	876/876	**2**	**99.89**	**876/876**	-	-	-
*bla*_TEM-1B_^2^	-	-	-	-	-	-	**3**	**100**	**861/861**	1	100	861/861
*floR* ^3^	-	-	-	-	-	-	**3**	**98.19**	**1214/1215**	-	-	-
*sul1* ^4^	1	100	840/840	1	100	840/840	1	100	840/840	-	-	-
*tet(A)* ^5^	1	100	1200/1200	1	100	1200/1200	1	100	1200/1200	1	100	1200/1200
**PlasmidFinder**	**S16BD08730**			**S18BD03394**			**S18BD05011**			**S18BD00684**		
**Replicon**	**Contig**	**Identity**	**Query/Template Length**	**Contig**	**Identity**	**Query/Template Length**	**Contig**	**Identity**	**Query/Template Length**	**Contig**	**Identity**	**Query/Template Length**
IncI2(Delta)	4	98.1	316/316	-	-	-	-	-	-	-	-	-
IncX4	5	99.73	374/374	-	-	-	-	-	-	-	-	-
Col440I	-	-	-	-	-	-	5	96.36	110/114	-	-	-
IncHI2	-	-	-	-	-	-	2	100	327/327	-	-	-
IncHI2A	-	-	-	-	-	-	2	99.52	630/630	-	-	-
IncX3	-	-	-	-	-	-	3	100	374/374	-	-	-
IncI1-I(Gamma)	-	-	-	-	-	-	-	-	-	2	100	142/142

In bold and underlined is indicated whether the AMR gene is located on a plasmid. Four chromosomal point mutations in the *parC* and *gyrA* genes conferring nalidixic acid and ciprofloxacin resistance were detected in each isolate. ^1^ Aminoglycoside; ^2^ beta-lactam; ^3^ phenicol; ^4^ sulfonamide; ^5^ tetracycline.

**Table 7:** Characteristics of isolates used in this study.

Isolate	Species	Conjugate	Information Known Before Sequencing
COL20160015	*E. coli*	no	contains *mcr-1* plasmid of >200 kb phenotypical resistance to AMP AMC PTZ TMO CTX CAZ FEP ETP MEM CIP GEN SMX TMP
R274	*E. coli*	plasmid from COL20160015	complete chromosome and contains *mcr-1* plasmid of >200 kb
S15BD05371	*S.* Typhimurium	no	contains *mcr-1* plasmid of >200 kb, phenotypical resistance to AMP COL CIP GEN SMX TET TMP
S16BD08730	*S.* Kentucky	no	phenotypical resistance to AMP CTX CIP GEN SMX TET
S18BD00684	*S.* Kentucky	no	phenotypical resistance to AMP CTX CAZ CHL CIP SMX TET TMP
S18BD03994	*S.* Kentucky	no	phenotypical resistance to AMP CTX CAZ CHL CIP GEN SMX TET TMP
S18BD05011	*S.* Kentucky	no	phenotypical resistance to AMP AZM CTX CAZ CHL CIP GEN SMX TET TMP

AMC = amoxicillin + clavulanate; AMP = ampicillin; AZM = azithromycin CAZ = ceftazidime; CHL = chloramphenicol; CIP = ciprofloxacin; COL = colistin; CTX = cefotaxime; ETP = ertapenem; FEP = cefepime; GEN = gentamicin; MEM = meropenem; PTZ = piperacillin + tazobactam; SMX = sulfamethoxazole; TMO = temocillin; TET = tetracycline; TMP = trimethoprim.

## Supplementary Materials

The following are available online at https://www.mdpi.com/2079-6382/9/8/503/s1: Figure S1: Mauve alignment between the reconstructed mcr-1 plasmid of assemblies 11–14. Figure S2: Visualization of hybrid assemblies of isolates COL20160015 and S15BD05371 from Flongle runs 1 (a), 2 (b), 3 (c), 4 (d) and 5 (e). Figure S3: Visualization of MinION assemblies of isolates COL20160015 and S15BD05371 from Flongle runs 1 (a), 2 (b), 3 (c), 4 (d) and 5 (e). Figure S4: Visualization of hybrid assemblies of isolates S16BD08730 (a), S18BD03994 (b), S18BD00864 (c) and S18BD05011 (d). Table S1: Antimicrobial resistance genes and plasmid replicons detected with ResFinder and PlasmidFinder in whole-genome MiSeq, MinION and hybrid assembly of isolate COL20160015 extracted with Genomic Tip 100. Table S2: Antimicrobial resistance genes and plasmid replicons detected with ResFinder and PlasmidFinder in whole-genome MiSeq, MinION and hybrid assembly of isolate R274 extracted with Genomic Tip 100. Table S3: Antimicrobial resistance genes and plasmid replicons detected with ResFinder and PlasmidFinder in whole-genome MiSeq, MinION and hybrid assembly of the conjugated isolate R274 extracted with Genomic Tip 100 with all chromosomal reads (mapped to CP000948.1) filtered out prior to assembly. Table S4: Quality parameters of the extracted DNA of isolate S15BD05371 used for sequencing Table S5: The most similar sequences from the NCBI nt database to the incomplete contigs in the hybrid assemblies. Table S6: Antimicrobial resistance genes detected with ResFinder in whole-genome MiSeq, MinION and hybrid assembly of isolate S15BD05371 extracted with MagCore. Table S7: Antimicrobial resistance genes detected with ResFinder in MiSeq, MinION and hybrid assembly of isolate S15BD05371 extracted with Genomic Tip500 with plasmid extraction buffers. Table S8: Antimicrobial resistance genes detected with ResFinder in MiSeq, MinION and hybrid assembly of isolate S15BD05371 extracted with a phenol chloroform plasmid extraction. Table S9: Antimicrobial resistance genes detected with ResFinder in MiSeq, MinION and hybrid assembly of isolate S15BD05371 extracted with phenol chloroform plasmid extraction followed up by an exonuclease digestion and amplification. Table S10: Antimicrobial resistance genes detected with ResFinder in the hybrid assembly of isolate COL20160015 from Flongle run 1. Table S11: Antimicrobial resistance genes detected with ResFinder in the hybrid assembly of isolate COL20160015 from Flongle run 2. Table S12: Antimicrobial resistance genes detected with ResFinder in the hybrid assembly of isolate COL20160015 from Flongle run 3. Table S13: Antimicrobial resistance genes detected with ResFinder in the hybrid assembly of isolate COL20160015 from Flongle run 4. Table S14: Antimicrobial resistance genes detected with ResFinder in the hybrid assembly of isolate S15BD05371 from Flongle run 5. Table S15: Statistics of all sequencing reads that were used in assemblies of this study.
