# Supplementary material for: Development of an NGS-Based Workflow for Improved Monitoring of Circulating Plasmids in Support of Risk Assessment of Antimicrobial Resistance Gene Dissemination
Source: Antibiotics (Basel). 2020 Aug 11;9(8):503. doi: 10.3390/antibiotics9080503 (PMC7460218; doi:10.3390/antibiotics9080503)
Supplement: Supplementary file 1 [file antibiotics-09-00503-s001.pdf]

Supplementary Materials

Development of an NGS-Based Workflow for Improved Monitoring of Circulating Plasmids in Support of Risk Assessment of Antimicrobial Resistance Gene Dissemination

Bas Berbers [1,2], Pieter-Jan Ceyssens [3], Pierre Bogaerts [4], Kevin Vanneste [1], Nancy H.C. Roosens [1], Kathleen Marchal [2,5,6] and Sigrid C.J. De Keersmaecker [1,*]

[1] Transversal Activities in Applied Genomics, Sciensano, 1050 Brussels, Belgium; Bas.Berbers@Sciensano.be (B.B.); Kevin.Vanneste@Sciensano.be (K.V.); Nancy.Roosens@Sciensano.be (N.H.C.R.)
[2] Department of Information Technology, IDLab, Ghent University, IMEC, 9000 Ghent, Belgium; Kathleen.Marchal@UGent.be
[3] Bacterial Diseases, Sciensano, 1050 Brussels, Belgium; Pieter-Jan.Ceyssens@Sciensano.be (P-J.C.)
[4] National Reference Center for Antimicrobial Resistance in Gram-Negative Bacteria, CHU UCL Namur, 5530 Yvoir, Belgium; Pierre.Bogaerts@uclouvain.be
[5] Department of Plant Biotechnology and Bioinformatics, Ghent University, 9000 Ghent, Belgium; Kathleen.Marchal@UGent.be
[6] Department of Genetics, University of Pretoria, 0002 Pretoria, South Africa; Kathleen.Marchal@UGent.be
* Correspondence: Sigrid.DeKeersmaecker@Sciensano.be; Tel.: +32-2-6425257

Received: 17 July 2020; Accepted: 7 August 2020; Published: date

Table S1. Antimicrobial resistance genes and plasmid replicons detected with ResFinder and PlasmidFinder in whole genome MiSeq, MinION and hybrid assembly of isolate COL20160015 extracted with Genomic Tip 100.

**ResFinder**	**MiSeq**			**MinION**			**Hybrid**		
**Resistance gene**	**Contig**	**Identity**	**Query / Template length**	**Contig**	**Identity**	**Query / Template length**	**Contig**	**Identity**	**Query / Template length**
aac(3)-IIa [1]	-	-	-	4	99.54	861 / 861	2	100	861 / 861
aac(3)-IId [1]	69	99.37	638 / 861	6	99.42	861 / 861	4	99.88	861 / 861
aac(3)-IId [1]	81	99.84	639 / 861	-	-	-	-	-	-
aac(6')-Ib-cr [1]	95	100	519 / 519	6	99.42	519 / 519	4	100	519 / 519
aadA1 [1]	72	100	792 / 792	4	99.75	792 / 792	2	100	792 / 792
aadA2b [1]	72	99.87	780 / 780	4	99.23	782 / 780	2	99.87	780 / 780

aadA5 [1]	114	100	789 / 789	**5**	**99.62**	**789 / 789**	**3**	**100**	**789 / 789**
aph(3'')-Ib [1]	110	100	803 / 804	**6**	**99.75**	**804 / 804**	**4**	**100**	**803 / 804**
aph(3'')-Ib [1]	-	-	-	**4**	**99.88**	**804 / 804**	**2**	**100**	**804 / 804**
aph(3')-Ia [1]	69	99.88	816 / 816	**4**	**99.27**	**817 / 816**	**2**	**99.88**	**816 / 816**
aph(6)-Id [1]	110	100	837 / 837	**4**	**99.52**	**837 / 837**	**4**	**99.88**	**837 / 837**
aph(6)-Id [1]	-	-	-	**6**	**99.76**	**837 / 837**	**2**	**100**	**837 / 837**
bla$_{KPC-2}$ [2]	**52**	**100**	**882 / 882**	**6**	**99.77**	**882 / 882**	**4**	**100**	**882 / 882**
bla$_{OXA-1}$ [2]	95	100	831 / 831	**6**	**99.28**	**831 / 831**	**4**	**100**	**831 / 831**
bla$_{TEM-1A}$ [2]	-	-	-	**4**	**99.88**	**862 / 861**	**2**	**99.77**	**861 / 861**
bla$_{TEM-1B}$ [2]	146	100	843 / 861	**6**	**99.88**	**862 / 861**	**4**	**100**	**861 / 861**
mcr-1.1 [3]	10	100	1626 / 1626	**4**	**99.88**	**1626 / 1626**	**2**	**100**	**1626 / 1626**
mdf(A) [4]	12	98.22	1233 / 1233	2	97.81	1233 / 1233	1	98.22	1233 / 1233
mph(A) [4]	93	100	906 / 906	**5**	**99.78**	**906 / 906**	**3**	**100**	**906 / 906**
mph(A) [4]	-	-	-	**6**	**99.89**	**906 / 906**	**4**	**99.67**	**922 / 921**
catA1 [5]	147	99.85	660 / 660	**5**	**98.94**	**660 / 660**	**3**	**99.85**	**660 / 660**
catB3 [5]	95	100	633 / 633	**6**	**99.53**	**635 / 633**	**4**	**100**	**633 / 633**
cmlA1 [5]	72	99.92	1260 / 1260	**4**	**99.76**	**1260 / 1260**	**2**	**99.92**	**1260 / 1260**
aac(6')-Ib-cr [6]	95	100	519 / 519	**6**	**99.42**	**519 / 519**	**4**	**100**	**519 / 519**
qnrB2 [6]	129	100	645 / 645	**6**	**99.22**	**645 / 645**	**4**	**100**	**645 / 645**
ARR-3 [7]	95	100	453 / 453	**6**	**100**	**453 / 453**	**4**	**100**	**453 / 453**
sul1 [8]	134	100	840 / 840	**6**	**99.64**	**840 / 840**	**4**	**99.88**	**840 / 840**
sul1 [8]	-	-	-	**5**	**99.76**	**840 / 840**	**3**	**100**	**840 / 840**
sul1 [8]	-	-	-	**6**	**99.76**	**840 / 840**	-	-	-
sul3 [8]	72	100	792 / 792	**4**	**99.5**	**794 / 792**	**2**	**100**	**792 / 792**

tet(A) [9]	32	100	1200 / 1200	**4**	**99.67**	**1200 / 1200**	**2**	**100**	**1200 / 1200**
dfrA17 [10]	114	100	474 / 474	**5**	**99.16**	**474 / 474**	**3**	**100**	**474 / 474**
dfrA19 [10]	91	100	570 / 570	**6**	**99.82**	**570 / 570**	**4**	**100**	**570 / 570**
**PlasmidFinder**	**MiSeq**			**MinION**			**Hybrid**		
**Replicon**	**Contig**	**Identity**	**Query / Template length**	**Contig**	**Identity**	**Query / Template length**	**Contig**	**Identity**	**Query / Template length**
Col156	89	98.03	152 / 154	-	-	-	8	98.03	152 / 154
IncFIB(AP001918)	50	98.97	682 / 682	5	98.09	682 / 682	3	99.17	603 / 682
IncFII	57	100	261 / 261	5	100	261 / 261	3	100	261 / 261
IncHI2	10	100	327 / 327	4	99.39	327 / 327	2	100	327 / 327
IncHI2A	10	99.52	630 / 630	4	99.05	630 / 630	2	99.52	630 / 630
IncN	52	99.42	514 / 514	6	99.03	514 / 514	4	99.42	514 / 514

In bold underlined are the AMR genes of which it is certain that they are located on a plasmid.

[1] Aminoglycoside, [2] Beta-lactam, [3] Colistin, [4] Macrolide, [5] Phenicol, [6] Quinolone, [7] Rifampicin, [8] Sulphonamide, [9] Tetracycline, [10] Trimethoprim

Table S2. Antimicrobial resistance genes and plasmid replicons detected with ResFinder and PlasmidFinder in whole genome MiSeq, MinION and hybrid assembly of isolate R274 extracted with Genomic Tip 100.

**ResFinder**	**MiSeq**			**MinION**			**Hybrid**		
**Resistance gene**	**Contig**	**Identity**	**Query / Template length**	**Contig**	**Identity**	**Query / Template length**	**Contig**	**Identity**	**Query / Template length**
aac(3)-IIa [1]	20	100	861 / 861	**2**	**99.3**	**862 / 861**	**2**	**100**	**861 / 861**
aadA1 [1]	20	100	792 / 792	**2**	**99.62**	**792 / 792**	**2**	**100**	**792 / 792**
aadA2b [1]	20	99.87	780 / 780	**2**	**99.87**	**780 / 780**	**2**	**99.87**	**780 / 780**
aph(3'')-Ib [1]	20	100	804 / 804	**2**	**99.88**	**804 / 804**	**2**	**100**	**804 / 804**
aph(3')-Ia [1]	20	99.88	816 / 816	**2**	**99.02**	**817 / 816**	**2**	**99.88**	**816 / 816**
aph(6)-Id [1]	20	100	837 / 837	**2**	**99.76**	**837 / 837**	**2**	**100**	**837 / 837**

bla$_{TEM-1A}$ [2]	20	100	861 / 861	**2**	**99.88**	**861 / 861**	**2**	**100**	**861 / 861**
mcr-1.1 [3]	78	100	1626 / 1626	**2**	**99.69**	**1629 / 1626**	**2**	**100**	**1626 / 1626**
mdf(A) [4]	2	99.92	1233 / 1233	1	99.59	1233 / 1233	1	99.92	1233 / 1233
cmlA1 [5]	20	99.92	1260 / 1260	**2**	**99.68**	**1261 / 1260**	**2**	**99.92**	**1260 / 1260**
sul3 [6]	20	100	792 / 792	**2**	**99.75**	**792 / 792**	**2**	**100**	**792 / 792**
tet(A) [7]	20	100	1200 / 1200	**2**	**99.58**	**1200 / 1200**	**2**	**100**	**1200 / 1200**
**PlasmidFinder**	**MiSeq**			**MinION**			**Hybrid**		
**Replicon**	**Contig**	**Identity**	**Query / Template length**	**Contig**	**Identity**	**Query / Template length**	**Contig**	**Identity**	**Query / Template length**
IncHI2	42	100	327 / 327	2	100	327 / 327	2	100	327 / 327
IncHI2A	25	99.52	630 / 630	2	99.21	630 / 630	2	99.52	630 / 630

In bold underlined are the AMR genes of which it is certain that they are located on a plasmid.

[1] Aminoglycoside, [2] Beta-lactam, [3] Colistin, [4] Macrolide, [5] Phenicol, [6] Sulphonamide, [7] Tetracycline

Table S3. Antimicrobial resistance genes and plasmid replicons detected with ResFinder and PlasmidFinder in whole genome MiSeq, MinION and hybrid assembly of the conjugated isolate R274 extracted with Genomic Tip 100 with all chromosomal reads (mapped to CP000948.1) filtered out prior to assembly.

**ResFinder**	**MiSeq**			**MinION**			**Hybrid**		
**Resistance gene**	**Contig**	**Identity**	**Query / Template length**	**Contig**	**Identity**	**Query / Template length**	**Contig**	**Identity**	**Query / Template length**
aac(3)-IIa [1]	2	100	861 / 861	1	99.19	864 / 861	1	100	861 / 861
aadA1 [1]	2	100	792 / 792	1	99.87	792 / 792	1	100	792 / 792
aadA2b [1]	2	99.87	780 / 780	1	99.74	781 / 780	1	99.87	780 / 780
aph(3'')-Ib [1]	2	100	804 / 804	1	99.88	804 / 804	1	100	804 / 804
aph(3')-Ia [1]	2	99.88	816 / 816	1	99.26	816 / 816	1	99.88	816 / 816
aph(6)-Id [1]	2	100	837 / 837	1	99.64	838 / 837	1	100	837 / 837

bla$_{\text{TEM-1A}}$ [2]	2	100	861 / 861	1	98.46	779 / 861	1	100	861 / 861
mcr-1.1 [3]	4	100	1626 / 1626	1	99.57	1631 / 1626	1	100	1626 / 1626
cmlA1 [4]	2	99.92	1260 / 1260	1	99.6	1260 / 1260	1	99.92	1260 / 1260
sul3 [5]	2	100	792 / 792	1	99.75	792 / 792	1	100	792 / 792
tet(A) [6]	2	100	1200 / 1200	1	99.5	1201 / 1200	1	100	1200 / 1200
**PlasmidFinder**	**MiSeq**			**MinION**			**Hybrid**		
**Plasmid replicon**	**contig**	**Identity**	**Query / Template length**	**Contig**	**Identity**	**Query / Template length**	**Contig**	**Identity**	**Query / Template length**
IncHI2	1	100	327 / 327	1	100	327 / 327	1	100	327 / 327
IncHI2A	1	99.52	630 / 630	1	99.21	630 / 630	1	99.52	630 / 630

[1] Aminoglycoside, [2] Beta-lactam, [3] Colistin, [4] Phenicol, [5] Sulphonamide, [6] Tetracycline

Table S4. Quality parameters of the extracted DNA of isolate S15BD05371 used for sequencing

**Extraction method**	**content extracted**	**Purity**		**Conc.**	**DIN**	**fragment size**	**qPCR**			**MinION**
		**260/280**	**260/230**	**ng/µl**	**score**	**bp**	**16S Cq**	***mcr-1* Cq**	**Cq difference***	**average read size**
G100 (extr-3)	whole genome	1.81	1.685	482.5	9.4	>60000	16.35	19.27	-2.92	7121
MagCore (extr-4)	whole genome	1.81	1.58	70.8	6.5	10758	14.63	17.05	-2.42	877
G500 (extr-5)	plasmid	1.76	1.21	263	9.1	>60000	14.13	16.37	-2.24	4946
G500-exo (extr-6)	plasmid	5.78	0.48	OOR [1]	OOR [2]	OOR [1]	18.84	20.96	-2.12	OOR [1]
phenol (extr-7)	plasmid	1.86	1.13	9	OOR [2]	>60000	20.23	17.19	3.04	842
phenol-ampli (extr-8)	plasmid	1.82	1.73	7.765	OOR [2]	31196	18.2	16.46	1.74	641

Each DNA extraction was performed multiple times, but only the quality values of the extracts that were sequenced are shown here. These quality values are representative to that of the replicates.*A positive Cq difference corresponds to a higher proportion of plasmid DNA than chromosomal DNA in the DNA extraction, and vice versa for a negative Cq difference.[1] DNA concentration was too low in G500-exo and excluded from further analyses. [2] the DNA concentration was too low to determine the DNA integrity (DIN).OOR = out of range.

Table S5. The most similar sequences from the NCBI nt database to the incomplete contigs in the hybrid assemblies.

DNA extraction	contig	top hit in NCBI nt database	description
G500 (as-13)	3	CP051269.1	*Salmonella enterica* subsp. *enterica* serovar Typhimurium strain OLF_FSR1_WB_Finch_ST-13 chromosome, complete genome
G500 (as-13)	4	CP051269.1	*Salmonella enterica* subsp. *enterica* serovar Typhimurium strain OLF_FSR1_WB_Finch_ST-13 chromosome, complete genome
G500 (as-13)	5	CP051269.1	*Salmonella enterica* subsp. *enterica* serovar Typhimurium strain OLF_FSR1_WB_Finch_ST-13 chromosome, complete genome
Phenol (as-14)	1	AP019374.1	*Salmonella enterica* subsp. *enterica* serovar 4,[5],12:i:- L-3838 DNA, complete genome
Phenol (as-14)	2	CP011428.1	*Salmonella enterica* subsp. *enteric*a strain YU39, complete genome
Phenol (as-14)	3	CP003836.1	*Salmonella enterica* subsp. *enterica* serovar Typhimurium str. U288, complete genome
Phenol (as-14)	4	LN999997.1	*Salmonella enterica* subsp. *enterica* serovar Typhimurium isolate SO4698-09 genome assembly, chromosome: I
Phenol (as-14)	6	LN999997.1	*Salmonella enterica* subsp. *enterica* serovar Typhimurium isolate SO4698-09 genome assembly, chromosome: I
Phenol (as-14)	7	AP019375.1	*Salmonella enterica* subsp. *enterica* serovar 4,[5],12:i:- L-3841 DNA, complete genome
Phenol (as-14)	8	AP019375.1	*Salmonella enterica* subsp. *enterica* serovar 4,[5],12:i:- L-3841 DNA, complete genome
Phenol (as-14)	9	CP019649.1	*Salmonella enterica* subsp. *enterica* serovar Typhimurium strain TW-Stm6 chromosome, complete genome
Phenol (as-14)	10	AP019374.1	*Salmonella enterica* subsp. *enterica* serovar 4,[5],12:i:- L-3838 DNA, complete genome
Phenol (as-14)	11	CP033257.1	*Salmonella enterica* subsp. *enterica* strain CFSA12 chromosome, complete genome
Phenol (as-14)	12	AP019374.1	*Salmonella enterica* subsp. *enteric*a serovar 4,[5],12:i:- L-3838 DNA, complete genome
Phenol (as-14)	13	AP014565.1	*Salmonella enterica* subsp. *enterica* serovar Typhimurium str. L-3553 DNA, complete genome
Phenol (as-14)	14	CP029568.1	*Salmonella enterica* strain DA34837 chromosome, complete genome
Phenol (as-14)	15	LN999997.1	*Salmonella enterica* subsp. *enterica* serovar Typhimurium isolate SO4698-09 genome assembly, chromosome: I
Phenol (as-14)	16	CP012681.1	*Salmonella enterica* subsp. *enterica* serovar Typhimurium strain 33676, complete genome
Phenol (as-14)	17	AP019374.1	*Salmonella enterica* subsp. *enterica* serovar 4,[5],12:i:- L-3838 DNA, complete genome
Phenol (as-14)	18	CP011428.1	*Salmonella enterica* subsp. *enterica* strain YU39, complete genome

Phenol (as-14)	19	AP019375.1	*Salmonella enterica* subsp. *enterica* serovar 4,[5],12:i:- L-3841 DNA, complete genome
Phenol (as-14)	20	AP019375.1	*Salmonella enterica* subsp. *enterica* serovar 4,[5],12:i:- L-3841 DNA, complete genome
Phenol (as-14)	21	AP019375.1	*Salmonella enterica* subsp. *enterica* serovar 4,[5],12:i:- L-3841 DNA, complete genome
Phenol (as-14)	22	AP019374.1	*Salmonella enterica* subsp. *enterica* serovar 4,[5],12:i:- L-3838 DNA, complete genome
Phenol (as-14)	23	AP014565.1	*Salmonella enterica* subsp. *enterica* serovar Typhimurium str. L-3553 DNA, complete genome
Phenol (as-14)	24	CP033255.1	*Salmonella enterica* subsp. *enterica* strain CFSA244 chromosome, complete genome
Phenol (as-14)	25	AP019375.1	*Salmonella enterica* subsp. *enterica* serovar 4,[5],12:i:- L-3841 DNA, complete genome
Phenol (as-14)	26	CP033257.1	*Salmonella enterica* subsp. *enterica* strain CFSA12 chromosome, complete genome
Phenol (as-14)	27	AP019375.1	*Salmonella enterica* subsp. *enterica* serovar 4,[5],12:i:- L-3841 DNA, complete genome
Phenol (as-14)	28	CP029567.1	*Salmonella enterica* strain DA34821 chromosome, complete genome
Phenol (as-14)	29	CP029568.1	*Salmonella enterica* strain DA34837 chromosome, complete genome
Phenol (as-14)	30	AP019375.1	*Salmonella enterica* subsp. *enterica* serovar 4,[5],12:i:- L-3841 DNA, complete genome
Phenol (as-14)	31	AP019375.1	*Salmonella enterica* subsp. *enterica* serovar 4,[5],12:i:- L-3841 DNA, complete genome
Phenol (as-14)	32	AP019375.1	*Salmonella enterica* subsp. *enterica* serovar 4,[5],12:i:- L-3841 DNA, complete genome
Phenol (as-14)	33	AP019375.1	*Salmonella enterica* subsp. *enterica* serovar 4,[5],12:i:- L-3841 DNA, complete genome
Phenol (as-14)	34	AP019375.1	*Salmonella enterica* subsp. *enterica* serovar 4,[5],12:i:- L-3841 DNA, complete genome
Phenol (as-14)	35	AP019375.1	*Salmonella enterica* subsp. *enterica* serovar 4,[5],12:i:- L-3841 DNA, complete genome
Phenol (as-14)	36	AP019375.1	*Salmonella enterica* subsp. *enterica* serovar 4,[5],12:i:- L-3841 DNA, complete genome
Phenol (as-14)	37	AP019375.1	*Salmonella enterica* subsp. *enterica* serovar 4,[5],12:i:- L-3841 DNA, complete genome
Phenol (as-14)	38	AP019375.1	*Salmonella enterica* subsp. *enterica* serovar 4,[5],12:i:- L-3841 DNA, complete genome
Phenol (as-14)	39	AP019375.1	*Salmonella enterica* subsp. *enterica* serovar 4,[5],12:i:- L-3841 DNA, complete genome
Phenol (as-14)	40	AP019375.1	*Salmonella enterica* subsp. *enterica* serovar 4,[5],12:i:- L-3841 DNA, complete genome
Phenol (as-14)	41	AP019375.1	*Salmonella enterica* subsp. *enterica* serovar 4,[5],12:i:- L-3841 DNA, complete genome
Phenol (as-14)	42	AP019375.1	*Salmonella enterica* subsp. *enterica* serovar 4,[5],12:i:- L-3841 DNA, complete genome
Phenol (as-14)	43	AP019375.1	*Salmonella enterica* subsp. *enterica* serovar 4,[5],12:i:- L-3841 DNA, complete genome
Phenol (as-14)	44	AP019375.1	*Salmonella enterica* subsp. *enterica* serovar 4,[5],12:i:- L-3841 DNA, complete genome

Phenol (as-14)	45	AP019375.1	*Salmonella enterica* subsp. *enterica* serovar 4,[5],12:i:- L-3841 DNA, complete genome
Phenol (as-14)	46	AP019375.1	*Salmonella enterica* subsp. *enterica* serovar 4,[5],12:i:- L-3841 DNA, complete genome
Phenol (as-14)	47	AP019374.1	*Salmonella enterica* subsp. *enterica* serovar 4,[5],12:i:- L-3838 DNA, complete genome
Phenol (as-14)	48	AP019375.1	*Salmonella enterica* subsp. *enterica* serovar 4,[5],12:i:- L-3841 DNA, complete genome
Phenol (as-14)	49	AP019375.1	*Salmonella enterica* subsp. *enterica* serovar 4,[5],12:i:- L-3841 DNA, complete genome
Phenol (as-14)	50	AP019375.1	*Salmonella enterica* subsp. *enterica* serovar 4,[5],12:i:- L-3841 DNA, complete genome
Phenol (as-14)	51	AP019375.1	*Salmonella enterica* subsp. *enterica* serovar 4,[5],12:i:- L-3841 DNA, complete genome
Phenol (as-14)	52	CP006717.1	*Salmonella enterica* subsp. *enterica* serovar Thompson str. RM6836, complete genome
Phenol (as-14)	53	AP019375.1	*Salmonella enterica* subsp. *enterica* serovar 4,[5],12:i:- L-3841 DNA, complete genome
Phenol (as-14)	54	AP019375.1	*Salmonella enterica* subsp. *enterica* serovar 4,[5],12:i:- L-3841 DNA, complete genome
Phenol (as-14)	55	HG326213.1	*Salmonella enterica* subsp. *enterica* serovar Typhimurium str. DT2, complete genome
Phenol (as-14)	56	AP019375.1	*Salmonella enterica* subsp. *enterica* serovar 4,[5],12:i:- L-3841 DNA, complete genome
Phenol (as-14)	57	CP020101.1	*Salmonella enterica* strain UFPRLABMOR1 chromosome, complete genome
Phenol (as-14)	58	AP019375.1	*Salmonella enterica* subsp. *enterica* serovar 4,[5],12:i:- L-3841 DNA, complete genome
Phenol (as-14)	59	AP019375.1	*Salmonella enterica* subsp. *enterica* serovar 4,[5],12:i:- L-3841 DNA, complete genome
Phenol (as-14)	60	CP024170.1	*Microbacterium* sp. Y-01 chromosome, complete genome
Phenol (as-14)	61	AP019375.1	*Salmonella enterica* subsp. *enterica* serovar 4,[5],12:i:- L-3841 DNA, complete genome
Phenol (as-14)	62	AP019375.1	*Salmonella enterica* subsp. *enterica* serovar 4,[5],12:i:- L-3841 DNA, complete genome

Table S6. Antimicrobial resistance genes detected with ResFinder in whole genome MiSeq, MinION and hybrid assembly of isolate S15BD05371 extracted with MagCore.

	MiSeq			MinION			Hybrid		
**Resistance gene**	**Contig**	**Identity**	**Query / Template length**	**Contig**	**Identity**	**Query / Template length**	**Contig**	**Identity**	**Query / Template length**
aac(3)-IV [1]	9	100	777 / 777	2	99.61	779 / 777	2	100	777 / 777
aac(6′)-Iaa [1]	1	100	438 / 438	1	100	438 / 438	1	100	438 / 438
aadA2b [1]	38	99.87	780 / 780	2	99.62	780 / 780	2	99.87	780 / 780
aph(4)-Ia [1]	9	100	1026 / 1026	2	100	1026 / 1026	2	100	1026 / 1026
bla$_{\text{TEM-1B}}$ [2]	9	100	861 / 861	2	99.54	862 / 861	2	100	861 / 861
mcr-1.1 [3]	9	100	1626 / 1626	2	99.82	1627 / 1626	2	100	1626 / 1626
lnu(F) [4]	38	100	761 / 822	2	99.61	761 / 822	2	100	761 / 822
qnrS1 [5]	51	100	657 / 657	2	100	657 / 657	2	100	657 / 657
sul3 [6]	40	100	792 / 792	2	99.87	792 / 792	2	100	792 / 792
tet(A) [7]	9	100	1200 / 1200	2	99.67	1200 / 1200	2	100	1200 / 1200
tet(B) [7]	31	100	1206 / 1206	1	99.92	1206 / 1206	1	100	1206 / 1206
dfrA12 [8]	38	100	498 / 498	2	99.6	498 / 498	2	100	498 / 498

[1] Aminoglycoside, [2] Beta-lactam, [3] Colistin, [4] Macrolide, [5] Quinolone, [6] Sulphonamide, [7] Tetracycline, [8] Trimethoprim.

Table S7. Antimicrobial resistance genes detected with ResFinder in MiSeq, MinION and hybrid assembly of isolate S15BD05371 extracted with Genomic Tip500 with plasmid extraction buffers.

	MiSeq			MinION			Hybrid		
**Resistance gene**	**Contig**	**Identity**	**Query / Template length**	**Contig**	**Identity**	**Query / Template length**	**Contig**	**Identity**	**Query / Template length**
aac(3)-IV [1]	1	100	777 / 777	3	99.87	777 / 777	2	100	777 / 777
aac(6′)-Iaa [1]	2	100	438 / 438	2	100	438 / 438	1	100	438 / 438
aadA2b [1]	290	99.87	780 / 780	3	99.74	780 / 780	2	99.87	780 / 780

aph(4)-Ia [1]	1	100	1026 / 1026	3	100	1026 / 1026	2	100	1026 / 1026
bla$_{\text{TEM-1B}}$ [2]	1	100	861 / 861	3	100	861 / 861	2	100	861 / 861
mcr-1.1 [3]	1	100	1626 / 1626	3	99.94	1626 / 1626	2	100	1626 / 1626
lnu(F) [4]	290	100	761 / 822	3	99.61	762 / 822	2	100	761 / 822
qnrS1 [5]	357	100	657 / 657	3	99.7	657 / 657	2	100	657 / 657
sul3 [6]	298	100	792 / 792	3	99.87	792 / 792	2	100	792 / 792
tet(A) [7]	1	100	1200 / 1200	3	99.58	1200 / 1200	2	100	1200 / 1200
tet(B) [7]	269	100	1206 / 1206	1	99.83	1206 / 1206	1	100	1206 / 1206
dfrA12 [8]	290	100	498 / 498	3	99.8	498 / 498	2	100	498 / 498

[1] Aminoglycoside, [2] Beta-lactam, [3] Colistin, [4] Macrolide, [5] Quinolone, [6] Sulphonamide, [7] Tetracycline, [8] Trimethoprim

Table S8. Antimicrobial resistance genes detected with ResFinder in MiSeq, MinION and hybrid assembly of isolate S15BD05371 extracted with a phenol chloroform plasmid extraction.

	**MiSeq**			**MinION**			**Hybrid**		
**Resistance gene**	**Contig**	**Identity**	**Query / Template length**	**Contig**	**Identity**	**Query / Template length**	**Contig**	**Identity**	**Query / Template length**
aac(3)-IV [1]	1	100	777 / 777	2	99.85	667 / 777	5	100	777 / 777
aac(6')-Iaa [1]	292	100	438 / 438	-	-	-	20	100	438 / 438
aadA2b [1]	321	99.87	780 / 780	2	99.87	780 / 780	5	99.87	780 / 780
aph(4)-Ia [1]	1	100	1026 / 1026	2	100	1026 / 1026	5	100	1026 / 1026
bla$_{\text{TEM-1B}}$ [2]	1	100	861 / 861	2	99.77	863 / 861	5	100	861 / 861
mcr-1.1 [3]	1	100	1626 / 1626	9	99.88	1626 / 1626	5	100	1626 / 1626
mcr-1.1 [3]	-	-	-	10	99.73	1102 / 1626	-	-	-
lnu(F) [4]	321	100	761 / 822	2	99.47	749 / 822	5	100	761 / 822
qnrS1 [5]	396	100	657 / 657	-	-	-	5	100	657 / 657
sul3 [6]	1	100	792 / 792	8	99.87	792 / 792	5	100	792 / 792

tet(A) [7]	1	100	1200 / 1200	2	99.75	1200 / 1200	5	100	1200 / 1200
tet(B) [7]	156	100	1206 / 1206	-	-	-	47	100	1206 / 1206
dfrA12 [8]	321	100	498 / 498	2	99.8	498 / 498	5	100	498 / 498

[1] Aminoglycoside, [2] Beta-lactam, [3] Colistin, [4] Macrolide, [5] Quinolone, [6] Sulphonamide, [7] Tetracycline, [8] Trimethoprim

Table S9. Antimicrobial resistance genes detected with ResFinder in MiSeq, MinION and hybrid assembly of isolate S15BD05371 extracted with phenol chloroform plasmid extraction followed up by an exonuclease digestion and amplification.

	**MiSeq**			**MinION**			**Hybrid**		
**Resistance gene**	**Contig**	**Identity**	**Query / Template length**	**Contig**	**Identity**	**Query / Template length**	**Contig**	**Identity**	**Query / Template length**
aac(3)-IV [1]	1	100	777 / 777	-	-	-	1	100	777 / 777
aadA2b [1]	2	99.87	780 / 780	-	-	-	1	99.87	780 / 780
aph(4)-Ia [1]	1	100	1026 / 1026	-	-	-	1	100	1026 / 1026
bla$_{TEM-1B}$ [2]	1	100	861 / 861	-	-	-	1	100	861 / 861
mcr-1.1 [3]	1	100	1626 / 1626	-	-	-	1	100	1626 / 1626
lnu(F) [4]	2	100	761 / 822	-	-	-	1	100	761 / 822
qnrS1 [5]	3	100	657 / 657	-	-	-	2	100	657 / 657
sul3 [6]	1	100	792 / 792	-	-	-	1	100	792 / 792
tet(A) [7]	1	100	1200 / 1200	-	-	-	1	100	1200 / 1200
dfrA12 [8]	2	100	498 / 498	-	-	-	1	100	498 / 498

[1] Aminoglycoside, [2] Beta-lactam, [3] Colistin, [4] Macrolide, [5] Quinolone, [6] Sulphonamide, [7] Tetracycline, [8] Trimethoprim

Table S10. Antimicrobial resistance genes detected with ResFinder in the Nanopore only and hybrid assembly of isolate COL20160015 from Flongle run 1.

	**Nanopore only**			**Hybrid**		
**Resistance gene**	**Contig**	**Identity**	**Query / Template length**	**Contig**	**Identity**	**Query / Template length**
aac(3)-IIa [1]	3	99.54	861 / 861	2	100	861 / 861
aac(3)-IId [1]	5	98.84	865 / 861	4	99.88	861 / 861
aac(6')-Ib-cr [1]	5	99.61	519 / 519	4	100	519 / 519
aadA1 [1]	3	99.87	792 / 792	2	100	792 / 792
aadA2b [1]	3	99.62	780 / 780	2	99.87	780 / 780
aadA5 [1]	4	99.62	789 / 789	3	100	789 / 789
aph(3'')-Ib [1]	5	99.5	806 / 804	4	99.88	804 / 804
aph(3'')-Ib [1]	3	99.75	805 / 804	2	100	804 / 804
aph(3')-Ia [1]	3	99.27	817 / 816	2	99.88	816 / 816
aph(6)-Id [1]	3	99.4	838 / 837	2	100	837 / 837
aph(6)-Id [1]	5	99.4	837 / 837	4	100	837 / 837
bla$_{KPC-2}$ [2]	5	99.32	883 / 882	4	100	882 / 882
bla$_{OXA-1}$ [2]	5	99.76	831 / 831	4	100	831 / 831
bla$_{TEM-1A}$ [2]	3	99.54	863 / 861	2	99.77	861 / 861
bla$_{TEM-1B}$ [2]	5	99.65	862 / 861	4	100	861 / 861
bla$_{TEM-156}$ [2]	3	99.54	863 / 861	-	-	-
mcr-1.1 [3]	3	99.82	1626 / 1626	2	100	1626 / 1626
mdf(A) [4]	1	97.88	1228 / 1233	1	98.22	1233 / 1233
mph(A) [4]	4	99.34	908 / 906	4	100	906 / 906
mph(A) [4]	5	99.89	906 / 906	3	100	906 / 906
catA1 [5]	4	99.09	660 / 660	3	99.85	660 / 660
catB3 [5]	5	99.84	633 / 633	4	100	633 / 633
cmlA1 [5]	3	99.6	1260 / 1260	2	99.92	1260 / 1260
aac(6')-Ib-cr [6]	5	99.61	519 / 519	4	100	519 / 519
qnrB2 [6]	5	99.07	646 / 645	4	100	645 / 645
ARR-3 [7]	5	99.78	453 / 453	4	100	453 / 453
sul1 [8]	5	99.52	829 / 828	4	100	840 / 840
sul1 [8]	5	99.64	840 / 840	3	100	840 / 840
sul1 [8]	4	99.76	840 / 840	4	100	840 / 840
sul3 [8]	3	99.5	794 / 792	2	100	792 / 792
tet(A) [9]	3	99.25	1201 / 1200	2	100	1200 / 1200
dfrA17 [10]	4	99.58	474 / 474	3	100	474 / 474
dfrA19 [10]	5	99.82	571 / 570	4	100	570 / 570

[1] Aminoglycoside, [2] Beta-lactam, [3] Colistin, [4] Macrolide, [5] Phenicol, [6] Quinolone, [7] Rifampicin, [8] Sulphonamide, [9] Tetracycline, [10] Trimethoprim

Table S11. Antimicrobial resistance genes detected with ResFinder in the Nanopore only and hybrid assembly of isolate COL20160015 from Flongle run 2.

	**Nanopore only**			**Hybrid**		
**Resistance gene**	**Contig**	**Identity**	**Query / Template length**	**Contig**	**Identity**	**Query / Template length**
aac(3)-IIa [1]	2	99.54	861 / 861	2	100	861 / 861
aac(3)-IId [1]	4	99.54	861 / 861	4	99.88	861 / 861
aac(6')-Ib-cr [1]	4	99.61	519 / 519	4	100	519 / 519
aadA1 [1]	2	99.5	793 / 792	2	100	792 / 792
aadA2b [1]	2	99.74	780 / 780	2	99.87	780 / 780
aadA5 [1]	3	99.62	789 / 789	3	100	789 / 789
aph(3'')-Ib [1]	4	99.61	509 / 804	4	99.88	804 / 804
aph(3'')-Ib [1]	2	99.5	804 / 804	2	100	804 / 804
aph(3')-Ia [1]	2	99.39	816 / 816	2	99.88	816 / 816
aph(6)-Id [1]	2	99.64	838 / 837	4	100	837 / 837
aph(6)-Id [1]	4	99.64	837 / 837	2	100	837 / 837
bla$_{KPC-2}$ [2]	4	99.89	882 / 882	4	100	882 / 882
bla$_{OXA-1}$ [2]	4	99.64	832 / 831	4	100	831 / 831
bla$_{TEM-1A}$ [2]	2	99.65	861 / 861	2	99.77	861 / 861
bla$_{TEM-1B}$ [2]	4	99.77	861 / 861	4	100	861 / 861
bla$_{TEM-156}$ [2]	2	99.65	861 / 861	-	-	-
mcr-1.1 [3]	2	99.82	1626 / 1626	2	100	1626 / 1626
mdf(A) [4]	1	97.57	1233 / 1233	1	98.22	1233 / 1233
mph(A) [4]	4	99.67	906 / 906	3	100	906 / 906
mph(A) [4]	3	99.89	906 / 906	4	100	906 / 906
catA1 [5]	3	98.94	661 / 660	3	99.85	660 / 660
catB3 [5]	4	99.37	633 / 633	4	100	633 / 633
cmlA1 [5]	2	99.6	1260 / 1260	2	99.92	1260 / 1260
aac(6')-Ib-cr [6]	4	99.61	519 / 519	4	100	519 / 519
qnrB2 [6]	4	99.07	645 / 645	4	100	645 / 645
ARR-3 [7]	4	99.78	453 / 453	4	100	453 / 453
sul1 [8]	4	99.64	840 / 840	3	100	840 / 840
sul1 [8]	3	99.4	840 / 840	4	100	840 / 840
sul1 [8]	4	99.29	840 / 840	4	100	840 / 840
sul3 [8]	2	99.75	792 / 792	2	100	792 / 792

tet(A) [9]	2	99.58	1201 / 1200	2	100	1200 / 1200
dfrA17 [10]	3	99.79	474 / 474	3	100	474 / 474
dfrA19 [10]	4	100	570 / 570	4	100	570 / 570

[1] Aminoglycoside, [2] Beta-lactam, [3] Colistin, [4] Macrolide, [5] Phenicol, [6] Quinolone, [7] Rifampicin, [8] Sulphonamide, [9] Tetracycline, [10] Trimethoprim.

Table S12. Antimicrobial resistance genes detected with ResFinder in the Nanopore only and hybrid assembly of isolate COL20160015 from Flongle run 3.

	**Nanopore only**			**Hybrid**		
**Resistance gene**	**Contig**	**Identity**	**Query / Template length**	**Contig**	**Identity**	**Query / Template length**
aac(3)-IIa [1]	2	98.61	862 / 861	2	100	861 / 861
aac(3)-IId [1]	5	99.3	861 / 861	4	99.88	861 / 861
aac(6')-Ib-cr [1]	5	99.23	520 / 519	4	100	519 / 519
aadA1 [1]	2	99.37	794 / 792	2	100	792 / 792
aadA2b [1]	2	98.85	783 / 780	2	99.87	780 / 780
aadA5 [1]	4	98.73	790 / 789	3	100	789 / 789
aph(3'')-Ib [1]	5	99.38	804 / 804	4	99.88	804 / 804
aph(3'')-Ib [1]	2	97.64	805 / 804	2	100	804 / 804
aph(3')-Ia [1]	2	98.9	816 / 816	2	99.88	816 / 816
aph(6)-Id [1]	5	99.28	838 / 837	4	100	837 / 837
aph(6)-Id [1]	2	97.5	840 / 837	2	100	837 / 837
bla$_{\text{KPC-2}}$ [1]	5	99.89	882 / 882	4	100	882 / 882
bla$_{\text{OXA-1}}$ [2]	5	99.15	591 / 831	4	100	831 / 831
bla$_{\text{TEM-1A}}$ [2]	2	99.08	868 / 861	2	99.77	861 / 861
bla$_{\text{TEM-1B}}$ [2]	5	99.88	861 / 861	4	100	861 / 861
bla$_{\text{TEM-156}}$ [2]	2	99.08	868 / 861	-	-	-
mcr-1.1 [3]	2	99.51	1629 / 1626	2	100	1626 / 1626
mdf(A) [4]	1	97.41	1236 / 1233	1	98.22	1233 / 1233
mph(A) [4]	4	99.23	906 / 906	3	100	906 / 906
mph(A) [4]	5	99.34	907 / 906	4	100	906 / 906
catA1 [5]	4	98.62	652 / 660	3	99.85	660 / 660
catB3 [5]	5	99.68	633 / 633	4	100	633 / 633
cmlA1 [5]	2	98.26	1262 / 1260	2	99.92	1260 / 1260
aac(6')-Ib-cr [6]	5	99.23	520 / 519	4	100	519 / 519
qnrB2 [6]	5	98.61	646 / 645	4	100	645 / 645
ARR-3 [7]	5	99.56	453 / 453	4	100	453 / 453
sul1 [8]	4	98.46	843 / 840	4	100	840 / 840

sul1 [8]	5	99.64	840 / 840	3	100	840 / 840
sul1 [8]	5	99.4	840 / 840	4	100	840 / 840
sul3 [8]	2	97.98	794 / 792	2	100	792 / 792
tet(A) [9]	2	98.75	1200 / 1200	2	100	1200 / 1200
dfrA17 [10]	4	98.32	477 / 474	3	100	474 / 474
dfrA19 [10]	5	99.65	570 / 570	4	100	570 / 570

[1] Aminoglycoside, [2] Beta-lactam, [3] Colistin, [4] Macrolide, [5] Phenicol, [6] Quinolone, [7] Rifampicin, [8] Sulphonamide, [9] Tetracycline, [10] Trimethoprim.

Table S13. Antimicrobial resistance genes detected with ResFinder in the Nanopore only and hybrid assembly of isolate COL20160015 from Flongle run 4.

	**Nanopore only**			**Hybrid**		
**Resistance gene**	**Contig**	**Identity**	**Query / Template length**	**Contig**	**Identity**	**Query / Template length**
aac(3)-IIa [1]	2	99.42	861 / 861	2	100	861 / 861
aac(3)-IId [1]	4	99.19	862 / 861	4	99.88	861 / 861
aac(3)-IId [1]	8	98.14	862 / 861	-	-	-
aac(6')-Ib-cr [1]	4	99	601 / 600	4	100	519 / 519
aac(6')-Ib-cr [1]	8	98	600 / 600	-	-	-
aadA1 [1]	2	99.5	795 / 792	2	100	792 / 792
aadA2b [1]	2	99.36	780 / 780	2	99.87	780 / 780
aadA5 [1]	3	99.62	789 / 789	3	100	789 / 789
aph(3'')-Ib [1]	7	98.26	806 / 804	4	99.88	804 / 804
aph(3'')-Ib [1]	4	99.13	804 / 804	2	100	804 / 804
aph(3'')-Ib [1]	8	98.51	806 / 804	-	-	-
aph(3'')-Ib [1]	4	99.5	805 / 804	-	-	-
aph(3'')-Ib [1]	7	98.26	806 / 804	-	-	-
aph(3'')-Ib [1]	2	99.75	804 / 804	-	-	-
aph(3')-Ia [1]	2	99.27	817 / 816	2	99.88	816 / 816
aph(6)-Id [1]	4	99.64	838 / 837	4	100	837 / 837
aph(6)-Id [1]	7	98.92	837 / 837	2	100	837 / 837
aph(6)-Id [1]	2	99.4	837 / 837	-	-	-
aph(6)-Id [1]	8	99.04	837 / 837	-	-	-
aph(6)-Id [1]	4	99.05	838 / 837	-	-	-
bla$_{KPC-2}$ [2]	4	99.66	882 / 882	4	100	882 / 882
bla$_{OXA-1}$ [2]	8	98.32	831 / 831	4	100	831 / 831
bla$_{OXA-1}$ [2]	4	99.52	832 / 831	-	-	-
bla$_{TEM-1A}$ [2]	2	99.77	862 / 861	2	99.77	861 / 861

*bla*TEM-1B [2]	4	99.42	865 / 861	2	99.88	861 / 861
*bla*TEM-156 [2]	2	99.77	862 / 861	-	-	-
mcr-1.1 [3]	2	99.94	1626 / 1626	2	100	1626 / 1626
mdf(A) [4]	1	97.73	1233 / 1233	1	98.22	1233 / 1233
mph(A) [4]	4	99.56	906 / 906	3	100	906 / 906
mph(A) [4]	7	99.67	906 / 906	4	100	906 / 906
mph(A) [4]	3	99.45	906 / 906	-	-	-
mph(A) [4]	8	99.34	906 / 906	-	-	-
mph(A) [4]	4	98.02	907 / 906	-	-	-
catA1 [5]	3	99.39	661 / 660	3	99.85	660 / 660
catA1 [5]	7	98.03	661 / 660	-	-	-
catB3 [5]	8	99.05	633 / 633	4	100	633 / 633
catB3 [5]	4	99.68	633 / 633	-	-	-
cmlA1 [5]	2	99.76	1260 / 1260	2	99.92	1260 / 1260
aac(6')-Ib-cr [6]	8	98	600 / 600	4	100	519 / 519
aac(6')-Ib-cr [6]	4	99	601 / 600	-	-	-
qnrB2 [6]	8	97.67	645 / 645	4	100	645 / 645
qnrB2 [6]	7	98.29	645 / 645	-	-	-
qnrB2 [6]	4	98.15	647 / 645	-	-	-
qnrB2 [6]	4	98.45	647 / 645	-	-	-
ARR-3 [7]	4	99.56	453 / 453	4	100	453 / 453
ARR-3 [7]	8	98.9	453 / 453	-	-	-
sul1 [8]	4	99.17	841 / 840	4	100	840 / 840
sul1 [8]	3	99.52	840 / 840	3	100	840 / 840
sul1 [8]	4	98.7	843 / 840	4	100	840 / 840
sul1 [8]	4	99.64	840 / 840	-	-	-
sul1 [8]	8	98.57	840 / 840	-	-	-
sul1 [8]	7	98.57	841 / 840	-	-	-
sul1 [8]	8	98.81	841 / 840	-	-	-
sul3 [8]	2	99.5	793 / 792	2	100	792 / 792
tet(A) [9]	2	99.58	1201 / 1200	2	100	1200 / 1200
dfrA17 [10]	3	99.79	474 / 474	3	100	474 / 474
dfrA18 [10]	8	99.12	570 / 570	-	-	-
dfrA19 [10]	7	99.3	572 / 570	4	100	570 / 570
dfrA19 [10]	8	99.12	570 / 570	-	-	-
dfrA19 [10]	4	99.13	573 / 570	-	-	-

dfrA19 [10]	4	99.48	572 / 570	-	-	-

[1] Aminoglycoside, [2] Beta-lactam, [3] Colistin, [4] Macrolide, [5] Phenicol, [6] Quinolone, [7] Rifampicin, [8] Sulphonamide, [9] Tetracycline, [10] Trimethoprim

Table S14. Antimicrobial resistance genes detected with ResFinder in the Nanopore only and hybrid assembly of isolate S15BD05371 from Flongle run 5.

	Nanopore only			Hybrid		
**Resistance gene**	**Contig**	**Identity**	**Query / Template length**	**Contig**	**Identity**	**Query / Template length**
aac(3)-IV [1]	2	*99.61*	777 / 777	2	100	777 / 777
aac(6')-Iaa [1]	1	*100*	438 / 438	1	100	438 / 438
aadA2b [1]	2	*99.49*	780 / 780	2	99.87	780 / 780
aph(4)-Ia [1]	2	*100*	1026 / 1026	2	100	1026 / 1026
*bla*TEM-1B [2]	2	*99.88*	861 / 861	2	100	861 / 861
mcr-1.1 [3]	2	*99.75*	1627 / 1626	2	100	1626 / 1626
lnu(F) [4]	2	*99.74*	761 / 822	2	100	761 / 822
qnrS1 [5]	2	*99.7*	657 / 657	2	100	657 / 657
sul3 [6]	2	*99.62*	795 / 792	2	100	792 / 792
tet(A) [7]	2	*99.33*	1202 / 1200	2	100	1200 / 1200
tet(B) [7]	1	*99.67*	1208 / 1206	1	100	1206 / 1206
dfrA12 [8]	2	*99.8*	498 / 498	2	100	498 / 498

[1] Aminoglycoside, [2] Beta-lactam, [3] Colistin, [4] Macrolide, [5] Quinolone, [6] Sulphonamide, [7] Tetracycline, [8] Trimethoprim

Table S15. Statistics of all sequencing reads that were used in assemblies of this study.

isolate	species	extraction method	sequencing technology	library preparation	used in following assemblies	Mean read length (bp)	Mean read quality	Median read length (bp)	Median read quality	Number of reads	Read length N50 (bp)	Total bases (bp)
COL201600015	*E. coli*	G100	MinION	SQK-LSK108 + EXP-NBD103 + fragmenting to 8kb	Minion and hybrid assembly COL20160015	7,758	9.7	7,566	10.0	75,322	10,110	584,335,770
R274	*E. coli*	G100	MinION	SQK-LSK108 + EXP-NBD103 + fragmenting to 8kb	Minion and hybrid assembly R274	6,455	8.7	6,327	9.0	47,812	7,755	308,620,023
S15BD05371	*S.* Typhimurium	G100	MinION	SQK-LSK108 + EXP-NBD103 + fragmenting to 8kb	as-6 + 11	7,121	8.7	6,874	8.9	95,008	8,503	676,580,030
S15BD05371	*S.* Typhimurium	MagCore	MinION	SQK-LSK108 + EXP-NBD103 + fragmenting to 8kb	as-7 + 12	758	9.5	398	9.7	772,993	989	585,584,477
S15BD05371	*S.* Typhimurium	G500	MinION	SQK-LSK108 + EXP-NBD103 +	as-8 + 13	4,946	8.6	4,432	8.8	102,176	7,461	505,396,084

				fragmenting to 8kb								
S15BD05371	*S.* Typhimurium	phenol	MinION	SQK-LSK108 + EXP-NBD103 + fragmenting to 8kb	as-9 + 14	842	8.8	610	8.9	112,647	1,107	94,864,356
S15BD05371	*S.* Typhimurium	phenol-ampli	MinION	SQK-LSK108 + EXP-NBD103 + fragmenting to 8kb	as-10 + 15	641	8.8	485	8.9	27,407	753	17,557,112
S16BD08730	*S.* Kentucky	MagCore	MinION	SQK-LSK108 + EXP-NBD103 + fragmenting to 8kb	S16BD08730 hybrid assembly	1,745	9.6	1,002	9.8	305,790	3,006	533,726,048
S18BD00684	*S.* Kentucky	MagCore	MinION	SQK-LSK108 + EXP-NBD103 + fragmenting to 8kb	S18BD00684 hybrid assembly	1,412	9.5	784	9.8	446,895	2,277	630,811,894
S18BD03994	*S.* Kentucky	MagCore	MinION	SQK-LSK108 + EXP-NBD103 + fragmenting to 8kb	S18BD03994 hybrid assembly	1,372	9.5	795	9.7	287,591	2,208	394,584,825

S18BD05011	*S.* Kentucky	MagCore	MinION	SQK-LSK108 + EXP-NBD103 + fragmenting to 8kb	S18BD05011 hybrid assembly	1,144	9.5	707	9.8	722,425	1,709	826,063,474
COL20160015	*E. coli*	G100	Flongle	SQK-LSK109 + fragmenting to 8kb	Flongle 1 hybrid + Nanopore only assembly	7,368	7.5	7,118	7.8	117,788	11,059	867,857,659
COL20160015	*E. coli*	G100	Flongle	SQK-LSK109	Flongle 2 hybrid + Nanopore only assembly	6,459	7.4	1,757	7.7	107,610	21,861	695,075,675
COL20160015	*E. coli*	G100	Flongle	SQK-LSK109 (LFB used)	Flongle 3 hybrid + Nanopore only assembly	4,334	6.2	1,343	6.2	114,758	14,190	497,339,098
COL20160015	*E. coli*	MagCore	Flongle	SQK-LSK109	Flongle 4 hybrid + Nanopore only assembly	7,189	7.2	3,483	7.6	100,353	15,787	721,405,028
S15BD05371	*S.* Typhimurium	MagCore	Flongle	SQK-LSK109	Flongle 5 hybrid + Nanopore only assembly	7,200	7.8	4,202	8.0	182,252	13,950	1,312,154,207
COL20160015	E. coli	G100	MiSeq	paired-end (forward reads)	MiSeq and hybrid assembly	219	34.4	250	37.2	709,981	250	155,613,734

					COL2016001 5 + Flongle 1-3 hybrid assembly							
COL2 01600 15	E. coli	G100	MiSeq	paired-end (reverse reads)	MiSeq and hybrid assembly COL2016001 5 + Flongle 1-3 hybrid assembly	219	32.3	250	35.9	709,981	250	155,608 ,764
R274	E. coli	G100	MiSeq	paired-end (forward reads)	MiSeq and hybrid assembly R274	249	31.7	251	33.4	315,054	251	78,281, 659
R274	E. coli	G100	MiSeq	paired-end (reverse reads)	MiSeq and hybrid assembly R274	249	22	251	21.5	315,054	251	78,275, 491
S15B D053 71	*S.* Typhi muriu m	G100	MiSeq	paired-end (forward reads)	as-1 + 11	248	32.4	251	35.3	369,346	251	91,472, 184
S15B D053 71	*S.* Typhi muriu m	G100	MiSeq	paired-end (reverse reads)	as-1 + 11	248	22.9	251	22.4	369,346	251	91,473, 638
S15B D053 71	*S.* Typhi muriu m	MagCor e	MiSeq	paired-end (forward reads)	as-2 + 12	228	33.9	250	36.9	790,045	250	180,418 ,288
S15B D053 71	*S.* Typhi	MagCor e	MiSeq	paired-end (reverse reads)	as-2+ 12	228	31	250	33	790,045	250	180,454 ,329

	murium											
S15BD05371	*S.* Typhimurium	G500	MiSeq	paired-end (forward reads)	as-3 + 13	247	32.7	251	35.7	137,178	251	33,832,661
S15BD05371	*S.* Typhimurium	G500	MiSeq	paired-end (reverse reads)	as-3 + 13	247	24.2	251	23.9	137,178	251	33,834,062
S15BD05371	*S.* Typhimurium	phenol	MiSeq	paired-end (forward reads)	as-4 + 14	242	33.7	251	36.6	334,020	251	80,947,612
S15BD05371	*S.* Typhimurium	phenol	MiSeq	paired-end (reverse reads)	as-4 + 14	242	25.5	251	25.1	334,020	251	80,980,749
S15BD05371	*S.* Typhimurium	phenol-ampli	MiSeq	paired-end (forward reads)	as-5 + 15	181	34.4	185	37.3	242,862	224	43,879,388
S15BD05371	*S.* Typhimurium	phenol-ampli	MiSeq	paired-end (reverse reads)	as-5 + 15	181	31.2	185	34.5	242,862	226	43,973,597
S16BD08730	*S.* Kentucky	MagCore	MiSeq	paired-end (forward reads)	S16BD08730 hybrid assembly	199	33.4	235	36.7	607,628	250	120,841,302
S16BD08730	*S.* Kentucky	MagCore	MiSeq	paired-end (reverse reads)	S16BD08730 hybrid assembly	199	31.6	236	34.5	607,628	250	120,940,255

S18BD00684	*S.* Kentucky	MagCore	MiSeq	paired-end (forward reads)	S18BD00684 hybrid assembly	202	34.4	226	37.3	619,910	248	125,053,848
S18BD00684	*S.* Kentucky	MagCore	MiSeq	paired-end (reverse reads)	S18BD00684 hybrid assembly	202	31.6	227	34.7	619,910	249	125,272,933
S18BD03994	*S.* Kentucky	MagCore	MiSeq	paired-end (forward reads)	S18BD03994 hybrid assembly	204	34.2	231	37.2	782,750	249	159,939,692
S18BD03994	*S.* Kentucky	MagCore	MiSeq	paired-end (reverse reads)	S18BD03994 hybrid assembly	205	31.7	233	34.8	782,750	250	160,156,721
S18BD05011	*S.* Kentucky	MagCore	MiSeq	paired-end (forward reads)	S18BD05011 hybrid assembly	242	33.4	251	36.4	674,244	251	163,445,295
S18BD05011	*S.* Kentucky	MagCore	MiSeq	paired-end (reverse reads)	S18BD05011 hybrid assembly	242	30.6	251	31.7	674,244	251	163,440,795
COL20160015	*E. coli*	MagCore	MiSeq	paired-end (forward reads)	Flongle 4 hybrid assembly	210	33.6	250	36.8	548,284	250	115,156,098
COL20160015	*E. coli*	MagCore	MiSeq	paired-end (reverse reads)	Flongle 4 hybrid assembly	210	31.2	250	33.8	548,284	250	115,235,485
S15BD05371	*S.* Typhimurium	MagCore	MiSeq	paired-end (forward reads)	Flongle 5 hybrid assembly	193	34.4	210	37.4	646,188	238	124,955,565
S15BD05371	*S.* Typhimurium	MagCore	MiSeq	paired-end (reverse reads)	Flongle 5 hybrid assembly	194	32.5	211	35.9	646,188	239	125,140,722

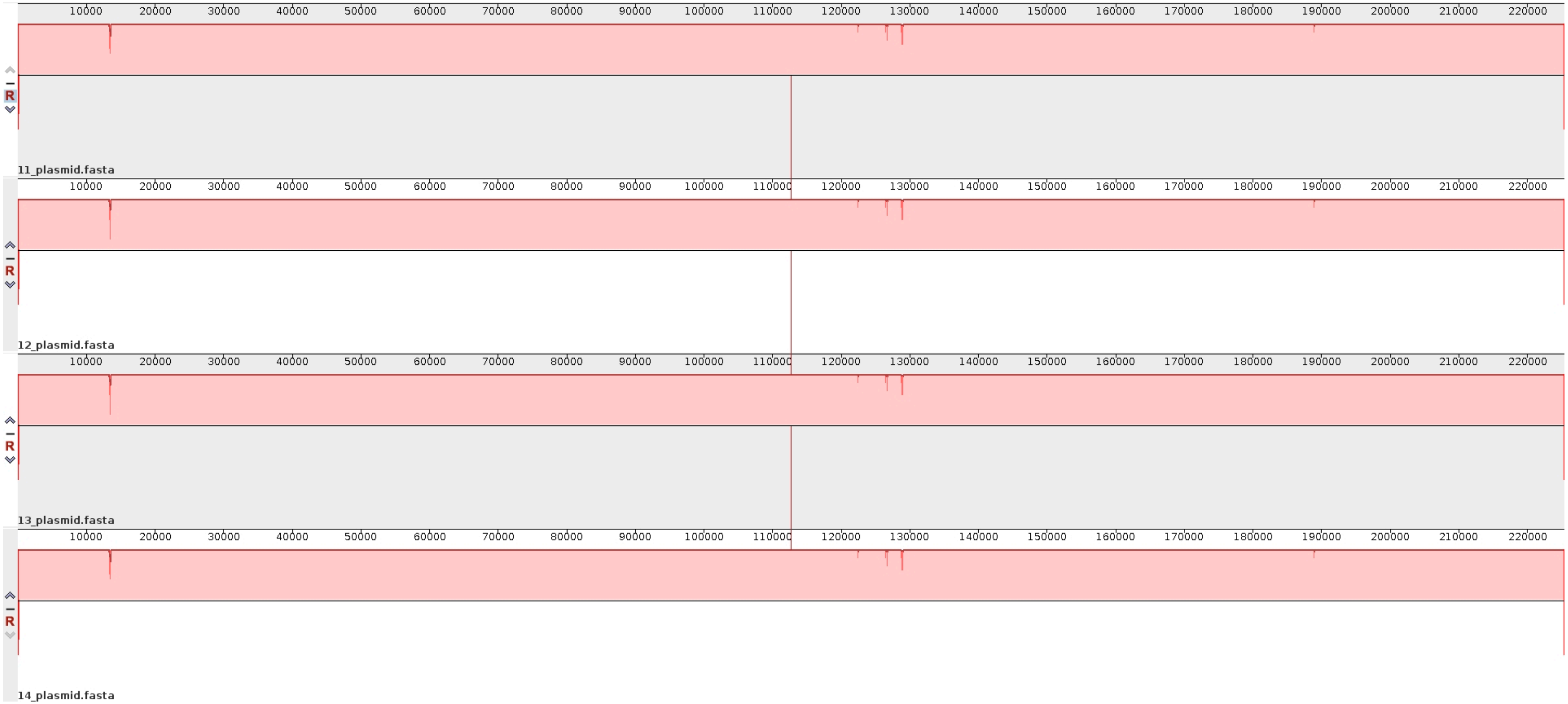

Figure S1. Mauve alignment between the reconstructed *mcr-1* plasmid of assemblies 11–14.

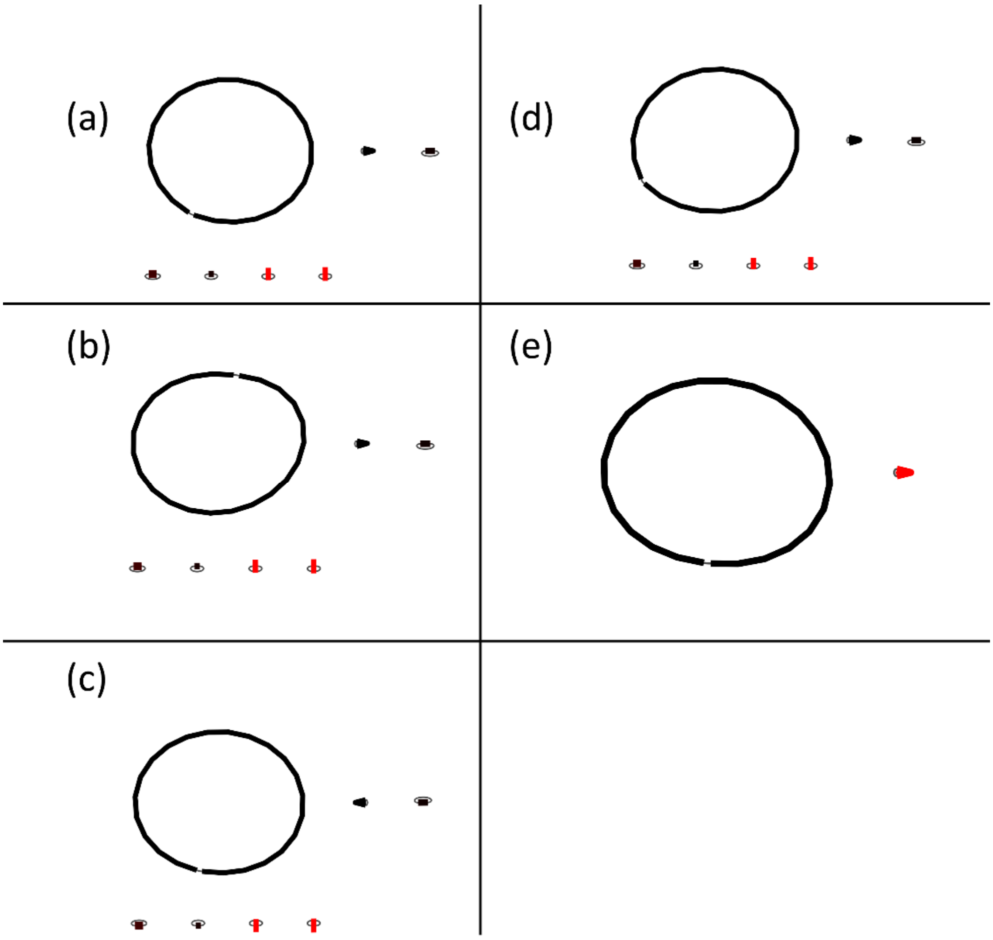

Figure S2. Visualization of hybrid assemblies of isolates COL20160015 and S15BD05371 from Flongle run **1 (a), 2 (b), 3 (c), 4 (d), 5 (e).**

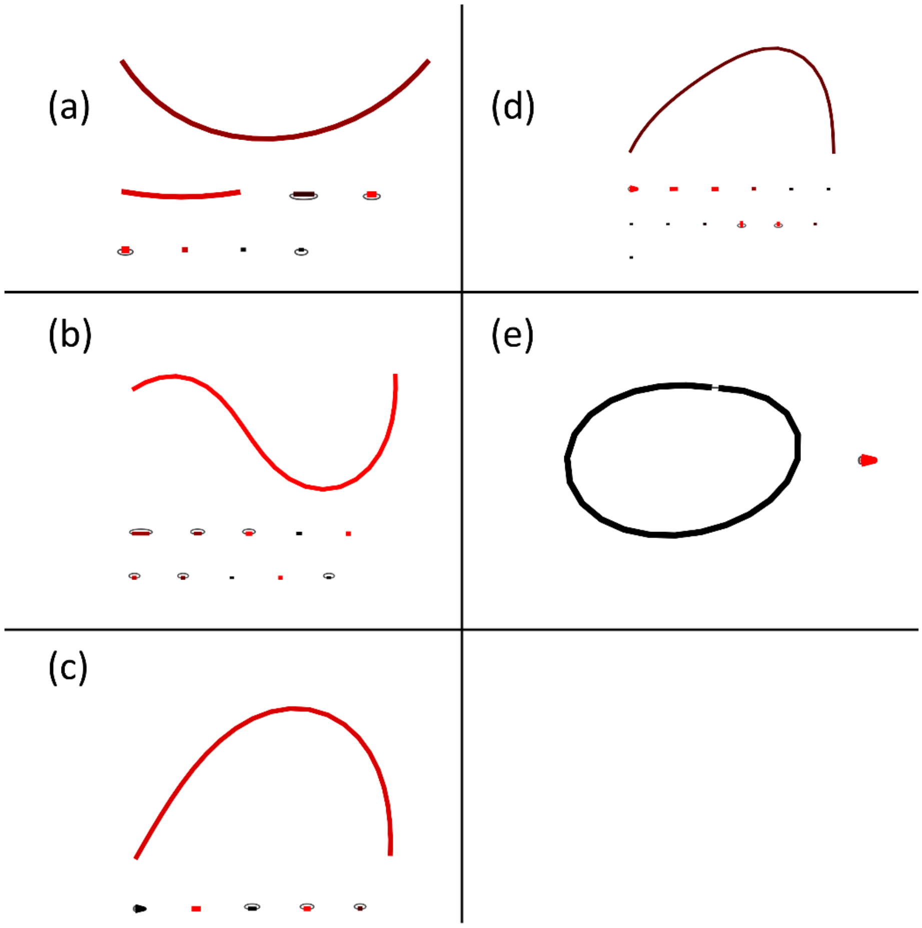

Figure S3. Visualization of MinION assemblies of isolates COL20160015 and S15BD05371 from Flongle run **1 (a), 2 (b), 3 (c), 4 (d), 5 (e).**

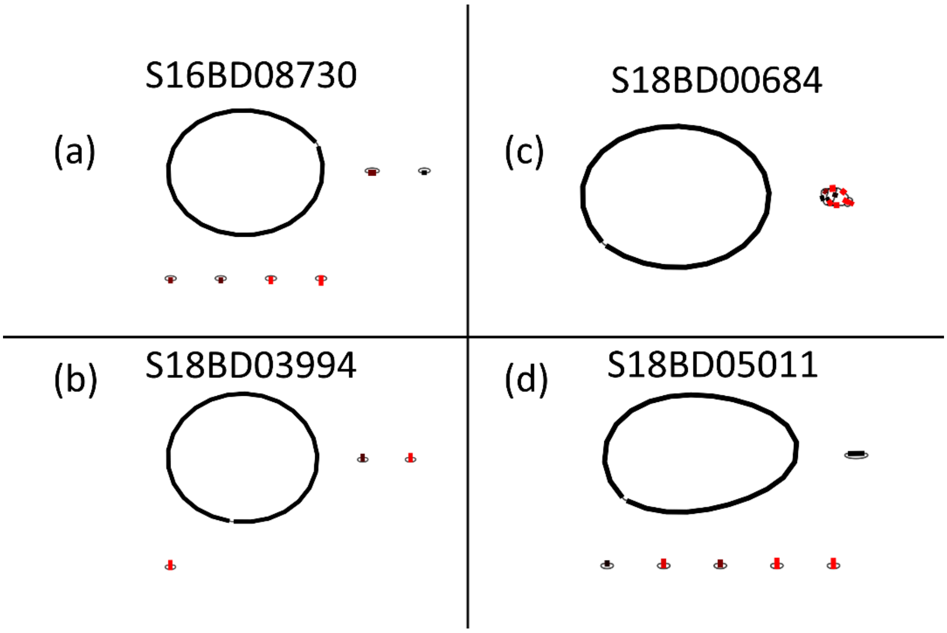

Figure S4. Visualization of hybrid assemblies of isolates S16BD08730 **(a),** S18BD03994 **(b),** S18BD00864 **(c)** and S18BD05011 **(d).**
